# Molecular Mechanisms of Glutamine Synthetase Mutations that Lead to Clinically Relevant Pathologies

**DOI:** 10.1371/journal.pcbi.1004693

**Published:** 2016-02-02

**Authors:** Benedikt Frieg, Boris Görg, Nadine Homeyer, Verena Keitel, Dieter Häussinger, Holger Gohlke

**Affiliations:** 1 Institute for Pharmaceutical and Medicinal Chemistry, Heinrich-Heine-University, Düsseldorf, Germany; 2 Clinic for Gastroenterology, Hepatology, and Infectious Diseases, Heinrich-Heine-University, Düsseldorf, Germany; Baltimore, UNITED STATES

## Abstract

Glutamine synthetase (GS) catalyzes ATP-dependent ligation of ammonia and glutamate to glutamine. Two mutations of human GS (R324C and R341C) were connected to congenital glutamine deficiency with severe brain malformations resulting in neonatal death. Another GS mutation (R324S) was identified in a neurologically compromised patient. However, the molecular mechanisms underlying the impairment of GS activity by these mutations have remained elusive. Molecular dynamics simulations, free energy calculations, and rigidity analyses suggest that all three mutations influence the first step of GS catalytic cycle. The R324S and R324C mutations deteriorate GS catalytic activity due to loss of direct interactions with ATP. As to R324S, indirect, water-mediated interactions reduce this effect, which may explain the suggested higher GS residual activity. The R341C mutation weakens ATP binding by destabilizing the interacting residue R340 in the *apo* state of GS. Additionally, the mutation is predicted to result in a significant destabilization of helix H8, which should negatively affect glutamate binding. This prediction was tested in HEK293 cells overexpressing GS by dot-blot analysis: Structural stability of H8 was impaired through mutation of amino acids interacting with R341, as indicated by a loss of masking of an epitope in the glutamate binding pocket for a monoclonal anti-GS antibody by L-methionine-*S*-sulfoximine; in contrast, cells transfected with wild type GS showed the masking. Our analyses reveal complex molecular effects underlying impaired GS catalytic activity in three clinically relevant mutants. Our findings could stimulate the development of ATP binding-enhancing molecules by which the R324S mutant can be repaired extrinsically.

## Author Summary

Glutamine synthetase (GS) catalyzes the ATP-dependent ligation of ammonia and glutamate to glutamine, which makes the enzyme essential for human nitrogen metabolism. Three mutations in human GS, R324C, R324S, and R341C, had been identified previously that lead to a glutamine deficiency, resulting in neonatal death in the case of R324C and R341C. However, the molecular mechanisms underlying this impairment of GS activity have remained elusive. Our results from computational biophysics approaches suggest that all three mutants influence the first step of GS’ catalytic cycle, namely ATP or glutamate binding. The analyses reveal a complex set of effects including the loss of direct interactions to substrates, the involvement of water-mediated interactions that alleviate part of the mutation effect, and long-range effects between the catalytic site and structural parts distant from it. As to the latter, experimental validation is in line with our prediction of a significant destabilization of helix H8 in the R341C mutant, which should negatively affect glutamate binding. Finally, our findings could stimulate the development of ATP-binding enhancing molecules for the R324S mutant, which has been suggested to have residual activity, that way extrinsically “repairing” the mutant.

## Introduction

Glutamine synthetase (GS, glutamate ammonia ligase, EC 6.3.1.2) catalyzes the ATP-dependent ligation of glutamate and ammonia to glutamine [1]. GS is ubiquitously expressed in human tissues. High expression levels of GS are found in astrocytes in brain tissues [2], where it is part of glutamate-glutamine cycling [3], and in perivenous hepatocytes, where it is part of the intercellular glutamine cycle and essential for ammonia detoxification by the liver [4–6]. Glutamate clearance, ammonia detoxification, and glutamine formation make GS essential for the human nitrogen metabolism [7, 8] and for neurological functionality. Accordingly, several links between changes in GS activity and neurological disorders have been described, including Alzheimer’s disease [9, 10], schizophrenia [11], hepatic encephalopathy [12–14] and epilepsy [15, 16]. In particular, two mutations in the GS gene (R324C in patient 1 and R341C in patient 2; throughout the manuscript, the sequence numbering of human GS is used) have been linked to congenital human GS deficiency with severe brain malformations resulting in multiorgan failure and neonatal death [17, 18]. In immortalized lymphocytes, R324C GS activity was reduced to about 12% of that found in wild type controls [17]. In fibroblasts from the father of patient 2, a 50% drop in specific GS activity was found, which may have been compensated for by a parallel increase in GS expression [17]. In a single case known to date, another GS mutation (R324S) was identified in a boy, now seven years old (patient 3), who is neurologically compromised due to the lack of ammonia detoxification and glutamine synthesis [19]. A plausible but not proven explanation for the survival of this patient would be the assumption of a higher level of GS residual activity compared to the other two GS mutants [20]. However, the molecular mechanisms for how these mutations lead to glutamine deficiency have not been understood.

**Fig 1:**
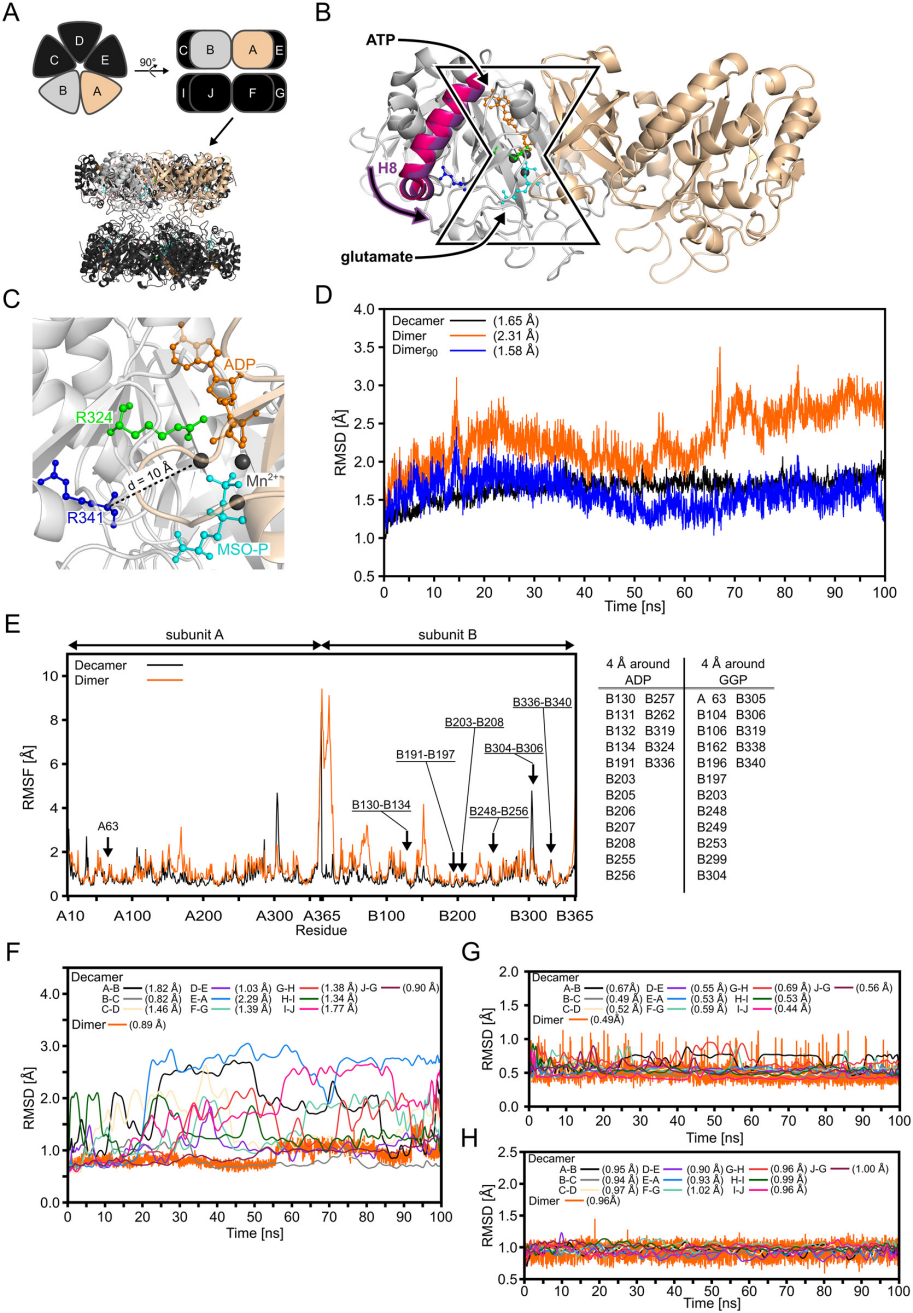
Structure of human GS, the dimeric model system, and the binding site in the crystal structure and during MD simulations. **(A):** Schematic representation of the GS decamer in top (top, left) and side (top, right) view. Subunits are labelled A to J. Below, the crystal structure of GS (PDB entry 2QC8 [22]) is shown in cartoon representation. Subunits A (beige) and B (grey) used for the dimeric model system are highlighted, as in the schematic representation. (**B):** Close-up view of the dimeric model system. Subunits A (beige) and B (grey) extracted from the GS decamer are shown in cartoon representation. ADP (orange) and the GS inhibitor L-methionine-*S*-sulfoximine phosphate (MSO-P, cyan) are depicted in ball-and-stick representation bound to the bifunnel-shaped catalytic site in the interface between two subunits; manganese ions (Mn^2+^) ions are shown as black spheres. ATP binding promotes a shift of helix 8 (H8; magenta from PDB entry 2UU7 of canine GS in the *apo* form [22]; violet from PDB entry 2QC8 of human GS bound to ATP and MSO-P [22]) that enables glutamate binding. (**C):** Close-up view of the binding site of GS in the crystal structure with ADP (orange), MSO-P (cyan), and both mutated residues [17, 18] R324 (green) and R341 (blue) in ball-and-stick representation. Mn^2+^ ions are shown as black spheres. Residue R341 is separated by ~ 10 Å from the center of the binding site (dashed line). (**D):** Backbone RMSD relative to the starting structure during 100 ns of MD simulations of the GS decamer (Decamer) and the dimeric model including all residues (Dimer) or only residues of the core region (Dimer_90_); the GS_ADP+GGP_ state was simulated. The core region comprises 90% of the residues with the lowest RMSF. Respective mean RMSD values are listed in brackets; SEM < 0.1 Å in all cases. **(E):** Residue wise RMSF for subunits A and B in the GS decamer and the dimeric model system during 100 ns of MD simulations of the GS_ADP+GGP_ state. The table lists residues that are separated by ≤ 4 Å from ADP or GGP; regions encompassing such residues are highlighted with an arrow and labeled in the figure. (**F):** Backbone RMSD of residues listed in the table in panel E relative to the starting structure during 100 ns of MD simulations for ten dimeric pairs in the GS decamer and the dimeric model. For the decamer, the backbone RMSD was plotted as smoothed cubic spline. Respective mean RMSD values are listed in brackets; SEM < 0.1 Å in all cases. (**G):** RMSD of ADP relative to the starting structure after superimpositioning of the protein atoms during 100 ns of MD simulations for ten dimeric pairs in the GS decamer and the dimeric model. For the decamer the RMSD was plotted as smoothed cubic spline. Respective mean RMSD values are listed in brackets; SEM < 0.1 Å in all cases. (**H):** RMSD of GGP relative to the starting structure after superimpositioning of the protein atoms during 100 ns of MD simulations for ten dimeric pairs in the GS decamer and the dimeric model. For the decamer the RMSD was plotted as smoothed cubic spline. Respective mean RMSD values are listed in brackets; SEM < 0.1 Å in all cases.

Human GS belongs to class II of GS enzymes [21] and forms a homodecamer [22] in which two pentameric rings stack to each other; a bifunnel-shaped catalytic site is located in each interface formed by two adjacent subunits, resulting in ten catalytic sites in total (Fig 1A and 1B). For glutamine formation by GS, a two-step mechanism has been suggested [23, 24]: In the first step adenosine triphosphate (ATP) binds to GS, which induces conformational changes to enable binding of glutamate [24]. After glutamate bound to the complex, the terminal phosphate group of ATP is transferred to the γ-carboxylate function of glutamate yielding adenosine diphosphate (ADP) and γ-glutamyl phosphate (GGP), a reactive acyl-phosphate intermediate. In the second step, an ammonium ion binds to a negatively charged pocket formed by D63, S66, Y162, and E305. The ammonium ion transfers a proton to D63 to yield ammonia. Subsequently, ammonia attacks GGP, which results in inorganic phosphate and a tetrahedral, positively charged reaction intermediate that is stabilized by E305 via a salt bridge interaction. E305 then gets protonated, which destabilizes the salt bridge, leading to the opening of the glutamate binding site and glutamine release.

Residue R324, which is mutated to cysteine (R324C) or serine (R324S), is located in the catalytic site and forms an ionic salt bridge with the β-phosphate group of ADP in the crystal structure (Fig 1C) [22]. It is reasonable to assume that R324 interacts analogously with the GS substrate ATP, although no crystal structure of human GS with ATP or an ATP analog is available. Residue R341 is located 10 Å away from the catalytic site (Fig 1C). No explanation has been put forward how the R341C mutation influences GS’ catalytic activity over that distance.

Here, we investigated changes in GS structure, dynamics, and energetics at the atomistic level due to the three GS mutations R324C, R324S, and R341C by molecular dynamics (MD) simulations, rigidity analysis [25, 26], and free energy calculations. Our data show direct effects of the R324C/S mutations on the ATP binding, which are attenuated in the case of R324S due to the emergence of water-mediated interactions to ATP. In contrast, for R341C, we demonstrate a long-range influence on both ATP and glutamate binding: First, R341 indirectly influences ATP binding as a stabilizing element in an amino acid triplet; second, R341 connects two topologically separated regions between which information transmission is essential for glutamate binding. *In vitro* studies on the GS mutant H281A-H284A-Y288A (HHY), predicted to mimic the loss of interactions in the R341C mutant, provide evidence for this influence. These results can semi-quantitatively explain the observed GS deficiencies linked to the three mutations [17–19] and provide a basis for investigations how to counteract the effect due to the R324S mutation.

## Materials and Methods

### Molecular dynamics simulations

We performed molecular dynamics (MD) simulations of the wild type GS and the three GS mutants, R324C, R342S, and R341C. Coordinates of human GS were obtained from a crystal structure available from the Protein Data Bank (PDB) [27] as PDB entry 2QC8 [22] solved at 2.6 Å resolution. Human GS is a homodecamer with ten identical subunits, each consisting of 373 amino acids. As MD simulations of the GS decamer are computationally highly expensive, we considered a dimeric model system containing only two adjacent subunits forming a single catalytic site. The dimeric model was generated by extracting two adjacent monomers from the GS crystal structure (chains A and B). The validity of the dimeric model was checked by comparative MD simulations of the GS wild type decamer and the GS wild type dimer. Both systems were simulated in the presence of bound ADP, the intermediate GGP, and magnesium ions (Mg^2+^).

Using the dimeric model we investigated the influence of the three mutations on four different states according to the suggested mechanism of glutamine formation [24]: GS without a ligand (GS_APO_), with bound ATP (GS_ATP_), with bound ATP and glutamate (GS_ATP+GLU_), and with bound ADP and GGP (GS_ADP+GGP_). All states were modelled for wild type GS and the three GS mutants R324C, R342S, and R341C. Models of GS mutants were obtained by amino acid exchanges in the wild type dimer using the SwissPDBViewer [28]. For all mutants the best ranked side chain rotamers were used as starting conformations.

The GS crystal structure contains non-covalently bound ADP, the inhibitor L-methionine-*S*-sulfoximine phosphate (MSO-P), manganese ions (Mn^2+^), chloride ions, and crystal water [22]. For the GS_ATP_ and GS_ATP+GLU_ states, ADP was changed to ATP by adding the missing atoms with the *LEaP* program [29] of AmberTools 1.4 [30] according to the library of Meagher *et al*. [31]. In the case of GS_ADP+GGP_, ADP coordinates were taken directly from the crystal structure, and hydrogen atoms were added according to the library of Meagher *et al* [31]. To generate GS_ATP+GLU_ and GS_ADP+GGP_, glutamate and GGP were manually modelled based on the coordinates of the structurally similar inhibitor MSO-P present in the crystal structure. Structurally bound Mn^2+^ ions were changed into Mg^2+^ ions, for which well-validated simulation parameters [32] are available. Moreover, GS is catalytically active with Mg^2+^ ions [33]. Magnesium ions were present in all states GS_APO_, GS_ATP_, GS_ATP+GLU_, and GS_ADP+GGP_ because the absence of divalent cations leads to a “relaxed” and inactive variant of GS [34, 35]. Nonetheless, we had to remove one Mg^2+^ ion in the case of GS_ATP_ and GS_ATP+GLU_ because the additional phosphate group of ATP causes clashes in the starting structure. Protonation states of histidines were assigned according to the protonation that was found to be most likely by visually inspecting the histidine environment.

The generated model systems were prepared for MD simulation with the *LEaP* program [29] of AmberTools 1.4 [30]. Sodium counter ions were added to the above described structures to neutralize each system. Model systems were placed in a truncated octahedral box of TIP3P water [36], leaving a distance of at least 11 Å between the solute and the border of the box. The finally obtained GS dimer systems comprised ~112,000 atoms. A system of the wild type GS decamer, prepared analogously, comprised ~354,000 atoms. For the polyphosphate chains of ADP and ATP, atomic partial charges and force field parameters were obtained from Meagher *et al*. [31]. Atomic partial charges for the substrate glutamate and the intermediate GGP were derived according to the restraint electrostatic potential fit (RESP) procedure [37]. Geometry optimizations and subsequent single point calculations were conducted with Gaussian03 [38] using the HF/6-31G* basis set. The resulting electrostatic potentials were fitted using *respgen* of AmberTools 1.4 [30]. Angle parameters for the phosphate group in GGP were taken from Homeyer *et al*. [39]. All other parameters were taken from the Amber ff99SB force field [40, 41].

The systems were relaxed by three steps of energy minimization, performed with the *sander* module of Amber11 [42]. First, harmonic restraints with a force constant of 5 kcal·mol^-1^·Å^-2^ were applied to all protein atoms, ligands, and structurally bound ions within the catalytic site while all other atoms were free to move (500 cycles steepest descent (SD) and 2000 cycles conjugate gradient (CG) minimization). Second, we reduced the harmonic restraints and applied a force constant of 1 kcal·mol^-1^·Å^-2^ (2000 cycles SD and 8000 cycles CG minimization). Finally, the positional restraints were removed completely, and all atoms were free to move (1000 cycles SD and 4000 cycles CG minimization).

The MD simulation procedure started by heating the respective system from 0 K to 100 K in a canonical (NVT) MD simulation of 50 ps length. During this heating step positional restraints of 1 kcal·mol^-1^·Å^-2^ were applied to all protein atoms, ligands, and structurally bound ions within the catalytic site. Afterwards, the temperature was raised from 100 K to ~300 K during 50 ps of isobaric-isothermal (NPT) MD. Subsequently, the density was adjusted to 1 g·cm^-3^ during 200 ps of NPT-MD. Finally, the harmonic positional restraints were removed by gradually decreasing the force constant from 1 to 0 kcal·mol^-1^·Å^-2^ in six NVT-MD runs of 50 ps length each. In the MD simulations, the particle mesh Ewald (PME) method [43–45] was employed to treat long-range electrostatic interactions. The SHAKE algorithm [46] was applied to all bonds involving hydrogens. A time step of 2 fs was used for the integration of the equations of motion. The distance cutoff for short range non-bonded interactions was set to 9 Å. In order to setup three independent MD production simulations, the target temperature was set to 299.9 K, 300.0 K, and 300.1 K in the equilibration, so that we obtained three different starting structures for subsequent MD production runs. Production MD simulations were performed in the NVT ensemble at 300 K for 100 ns. Coordinates were saved in a trajectory file every 20 ps. Using this MD simulation protocol, we generated three independent MD simulations for four different states (see above), for wild type GS and the GS mutants R324C, R324S, and R341C, which resulted in 3 × 4 × 4 = 48 MD simulations and an aggregate simulation time of 4.8 μs.

The 20–100 ns interval of each production run was considered for analysis. The analysis of the MD trajectories was carried out with *ptraj* [47] of AmberTools 1.4 [30]. The following measures were computed: the root mean-square fluctuation (RMSF) as a measure of mobility, the root mean-square deviation (RMSD) as a measure of structural similarity, the average secondary structure along the MD trajectory, water density grids, and distances. In addition, hydrogen bond interactions were determined using a distance of 2.8 Å between the two donor and acceptor atoms and an angle (donor atom, H, acceptor atom) of 120° as cutoff criteria for strong hydrogen bonds, and a distance of 3.2 Å and an angle of 120° as cutoff criteria for weak hydrogen bonds [48]. We analyzed whether a water-mediated chain of hydrogen bonds exists between ATP and C324 or S324, respectively. A water-mediated interaction was considered present when all hydrogen bonds in the water chain fulfilled the above distance and angle criteria. Results from three independent trajectories of the same system are expressed as means ± standard error of the mean (SEM). Results were analyzed with the R software [49] using the two-sided Student’s *t*-test. *P* values < 0.05 were considered significant.

### Constraint network analysis

The Constraint Network Analysis (CNA) approach allows linking biomacromolecular structure, flexibility, (thermo-)stability, and function [26]. To analyze the effect of R341 on the structural stability of GS, we extracted an ensemble of 4000 structures from the 20–100 ns interval of the MD simulation of wild type GS in the GS_ADP+GGP_ state that was equilibrated at 300.1 K; in this interval, the RMSD of GS relative to the starting structure remained particularly stable on average (~ 2 Å). In addition, we extracted an ensemble of 400 equally distributed structures from the 20–100 ns interval of MD simulations of the decameric wild type GS in the GS_ADP+ADP_ state. Coordinates (excluding water molecules, ions, and ligands) were extracted by *mm_pbsa*.*pl* [50] of Amber 11 [42]. Coordinates of an R341A GS mutant, used to mimic the loss of interactions of the R341 side chain with its environment, were generated employing the Ala-scan functionality of *mm_pbsa*.*pl*. This led to two sets of coordinates for dimeric and decameric GS, respectively, that differed only in residue 341. With CNA, thermal unfolding simulations of wild type GS and R341A GS were then performed to identify differences in the GSs’ structural stability [51]. For this, a hydrogen bond energy cutoff in the range of 0 to 6 kcal·mol^-1^ with steps of 0.1 kcal·mol^-1^ was used [26]. Stability maps [51] were then generated, which report when a rigid contact between two amino acids *i* and *j* (*rc*_*ij*_) vanishes during the thermal unfolding simulation [25]. Finally, a difference stability map was calculated as *rc*_*ij*_(wild type GS)–*rc*_*ij*_(R341A GS); differences with *p* < 0.05 according to a Welch test [52] were considered significant.

### Computation of effective binding energies

Effective binding energies, i.e., the sum of gas-phase energies plus solvation free energies [53, 54], for the substrates ATP and glutamate were computed by the molecular mechanics Poisson-Boltzmann surface area (MM-PBSA) approach [55, 56]. The computations were performed with the *mm_pbsa*.*pl* script [50] of Amber 12 [57], using the ff99SB force field [40, 41] as in the MD simulations. The polar part of the solvation free energy was computed with the PBSA solver implemented in Amber 12 using dielectric constants of 4 and 80 for the solute and the solvent, respectively, and Parse radii [58] for the solute atoms. A solute dielectric constant of 4 was recommended for highly charged binding sites of proteins [59, 60], as given in the case of GS [22], to adequately account for screening effects of the binding site region.

Effective binding energies were computed according to the 1-trajectory MM-PBSA approach, in which snapshots of complex, receptor, and ligand are obtained from MD simulation of the complex [55]. While this approach neglects energetic effects due to conformational changes upon binding, it generally results in lower statistical uncertainties [55]. Contributions due to changes in the configurational entropy of the ligand or the receptor upon complex formation were neglected, too, in order to avoid introducing additional uncertainty in the computations [53, 59, 61]. Conformational ensembles for the computations were generated by extracting 4000 snapshots from the 20–100 ns interval of the MD trajectories of the GS_ATP_ and GS_ATP+GLU_ states of wild type GS and all three mutants R324C, R324S, and R341C. In the case of the GS_ATP+GLU_ state, effective binding energy calculations were performed considering glutamate as the ligand, whereas ATP was considered part of the receptor. The effective binding energies were averaged over the respective ensembles.

Relative effective binding energies (ΔΔ*G*) were calculated by subtracting the effective binding energy of the wild type (Δ*G*_wild type_) from the effective binding energy of the mutant Δ*G*_mutant_ for trajectory {1, 2, 3} (eq 1).

$$\Delta \Delta G_{\left\{1,\;2,\;3\right\}}=\Delta G_{\text{mutant},\left\{1,\;2,\;3\right\}}-\Delta G_{\text{wild type},\left\{1\;,2,\;3\right\}}$$ (1)

Results from the three independent MD simulations for a system are expressed as mean over the ΔΔ*G*_{1, 2, 3}_. The SEM over the three independent MD simulations for a system *X* (*SEM*_*X*_) was calculated by error propagation according to eq 2.

$$SEM=\;\sqrt{SEM_{1}{}^{2}+\;SEM_{2}{}^{2}+\;SEM_{3}{}^{2}}$$ (2)

where *SEM*_{1,2,3}_ is the SEM for trajectory {1, 2, 3}. The SEM of the relative effective binding energy (eq 2) was calculated according to eq 3.

$$SEM_{total}=\;\sqrt{SEM_{mutant}{}^{2}+\;SEM_{wild\;type}{}^{2}}$$ (3)

A one-sample *t*-test with ΔΔ*G* = 0 as reference was performed using the R software [49]. *P* values < 0.05 were considered significant.

### Materials

L-methionine-*S*-sulfoximine (MSO) and polyclonal antibodies raised against the C-terminus of glutamine synthetase were from Sigma (Deisenhofen, Germany). The monoclonal antibody directed against GS (clone 6) was from Beckton-Dickinson (Heidelberg, Germany). The monoclonal antibody against GFP (green fluorescent protein), which cross-reacts with the YFP-variant (yellow fluorescent protein), was from Miltenyi-Biotech (Bergisch-Gladbach, Germany). Horseradish peroxidase-coupled goat anti-mouse IgG antibodies were from Bio-Rad International (Munich, Germany). Horseradish peroxidase-coupled goat anti-rabbit IgG antibodies were from Dako (Eching, Germany). The monoclonal antibody against glyceraldehyde-3-phosphate dehydrogenase (GAPDH) was from Biodesign International (Cologne, Germany). Lipofectamine 2000 was from Life Technologies (Darmstadt, Germany).

### Culturing and experimental treatment of HEK293 cells

Human embryonic kidney 293 (HEK293) cells were cultured on Petrie dishes (diameter = 60 mm) in minimal essential medium (MEM) containing Earle´s salt, L-glutamine and 5% fetal bovine serum (PAA, Linz, Austria). HEK293 cells were grown to about 70% confluency before cDNA (2 μg/dish) was introduced by lipofection using Lipofectamin 2000 according to the manufacturer’s instructions. 24 h after transfection, cells were either treated with L-methionine-*S*-sulfoximine (MSO, 3 mmol/l) or were left untreated for 2 h.

### Cloning and site-directed mutagenesis of human GS

Human glutamine synthetase was cloned using human liver cDNA and the following primers GS-YFP-for: 5’-CGGAATTCATGACCACCTCAGCAAGTTC-3’ and GS-YFP-rev: 5’-CGGGATCCGCGTAATTTTTGTACTGGAAGG-3’. The forward primer contained an EcoRI restriction site, and the stop codon in the reverse primer was replaced by a BamHI site. The PCR product was cloned into the pEYFP-N1 vector (Clontech, Palo Alto, CA). Mutations were introduced into WT-GS-YFP using the QuikChange Multi Site-Directed Mutagenesis Kit (Agilent Technologies, Santa Clara, USA) and the following mutagenesis primers GS-R341A: 5’-GGTTACTTTGAAGATCGTGCCCCCTCTGCCAACTGCG-3’ and GS-SKR: 5’-GGCCATTGAGAAACTAGCCGCGGCGCACCAGTACCACATCC-3’. For the HHY variant the mutations were introduced sequentially using the following primers: GS-H281/84A-for: 5’-GAGAAACTAAGCAAGCGGGCCCAGTACGCCATCCGTGCCTATGATCC-3’ and GS-Y288A: 5’-CAGTACGCCATCCGTGCCGCTGATCCCAAGGGAGGCCTGG-3’. Successful cloning and mutagenesis was verified by sequencing (GenBank accession number: NM_002065).

### Western- and dot-blot analysis

Western-blot analysis was performed as described recently [62]. In brief, at the end of the experimental procedure proteins were purified from HEK293 cells and protein content was determined by the BioRad protein assay (BioRad, Munich, Germany). After polyacrylamide gel electrophoresis (10%), proteins were transferred onto nitrocellulose membranes using a semi-dry blotting chamber (BioRad, Munich, Germany). Membranes were incubated in bovine serum albumin (BSA, 10%) for 30 min and incubated with antibodies against GS (mAb, 1:5,000; pAb, 1:5,000), green fluorescent protein (GFP mAb 1:5,000), or glyceraldehyde-3-phosphate dehydrogenase (GAPDH, mAb, 1:5,000). Primary antibodies were detected using horseradish peroxidase-coupled anti-mouse or anti-rabbit IgG antibodies (1:10,000, 2 h at room temperature), respectively.

Dot-blot analysis was performed as described recently [62, 63] by spotting 2 μg of protein in a volume of 2 μl protein lysis buffer on a nitrocellulose membrane. After spots were dried for 30 min. at room temperature, immunodetection was performed using the membrane as described above for Western-blot analysis.

Peroxidase activity on the membranes was detected using Western-Lightning chemiluminescence reagent plus (Perkin Elmer, Waltham, USA). Digital images were captured using the Kodak Image Station 4000MM. Signal intensities were measured by densitometric analysis using the Kodak Molecular Imaging Software.

### Immunofluorescence analysis

Immunofluorescence analysis was performed by confocal laserscanning microscopy (LSM510-META (Carl Zeiss AG, Oberkochem, Germany). HEK293 cells were seeded on MaTek dishes (MatTek Corporation, Ashland, USA) and transfected with cDNA constructs as described above. At the end of the transfection procedure, cells were incubated with Hoechst34580 (1:10,000; Life Technologies, Darmstadt, Germany) for 10 min at 37°C in an incubator (5% CO_2_). Cells were washed twice with phosphate-buffered saline before MatTek dishes were mounted on the LSM510-META and analyzed for YFP and Hoechst34580 immunofluorescence.

### Analysis of results

For statistical analysis, experiments were carried out with three separate HEK293 seedings. Results are expressed as mean values ± SEM and compared using a two-sided Student’s *t*-test (Excel for Windows; Microsoft, Redmond, USA). *P* values < 0.05 were considered significant.

## Results

### Validation of the dimeric model system

In order to perform MD simulations more efficiently and, thus, improve conformational sampling of wild type GS and GS mutants, we established a model system consisting of two adjacent subunits of the GS decamer forming a single catalytic site (Fig 1B and 1C). The dimeric model system and the decameric structure of wild type GS in the GS_ADP+GGP_ state were subjected to MD simulations of 100 ns length at *T* = 300 K to probe the stability of the systems and to validate that the binding site structure does not deteriorate in the model system.

First, the RMSD, a measure of structural deviation along the MD trajectories, of all protein backbone atoms relative to the starting structure was analyzed (Fig 1D). The RMSD values for both systems increase until 20 ns. After this period, the RMSD of the GS decamer remains constant and below 2.0 Å (mean RMSD over 100 ns: 1.65 Å (SEM < 0.1 Å)). Thus, the structure of the GS decamer shows only minor changes during the MD simulation. The dimeric model system yields a mean RMSD of 2.31 Å (SEM < 0.1 Å), with a maximal value of ~3.5 Å after 70 ns (Fig 1D). The overall structural change of the dimeric model system is slightly larger than that of the GS decamer. Still, these values are in the range observed for other protein systems of that size during MD simulations of that length [64, 65]. Regions in the dimeric model that show particularly large conformational variations were identified by computing the RMSF per residue; the RMSF is a measure of the average atomic mobility. The largest differences in the conformational variability between the dimeric model and the GS decamer occur at the N- and C-termini of the subunits (Fig 1E). The lower RMSF values for the GS decamer result from all terminal protein chains interacting with adjacent subunits; such interactions are missing for the termini of the dimeric model. Hence, when considering only those 90% of the residues with the lowest RMSF (“core region”) in the RMSD calculations, the mean RMSD of the dimeric model decreases to 1.58 Å (SEM < 0.1 Å) (Fig 1D, Dimer_90_), which is comparable to the value for the GS decamer (see above).

Second, we focused our analysis on the binding site region, i.e., all residues that are within 4 Å of the bound ADP and GGP (Fig 1E). These residues show low mean RMSD values relative to the starting structure of 0.89 Å (SEM < 0.1 Å) in the MD simulations of the dimeric model system and for the GS decamer in the range of 0.82–2.29 (SEM < 0.1 Å) (Fig 1F). Regarding the bound ligands themselves, mean RMSD for ADP (GGP) (Fig 1G and 1H) of 0.44–0.69 Å (0.90–1.03 Å) in the case of the decamer and 0.49 Å (0.96 Å) in the case of the dimeric model were found (SEM < 0.1 Å in all cases).

In summary, small structural deviations of similar magnitude are found for the core regions, the binding sites, and the ligands of both the dimeric model system and the GS decamer with respect to the starting structures, demonstrating that both systems remain structurally stable over the simulation time. The dimeric model will thus be used to investigate the effects of the GS mutations.

### Effects of mutations of R324 on interactions to ATP

**Fig 2:**
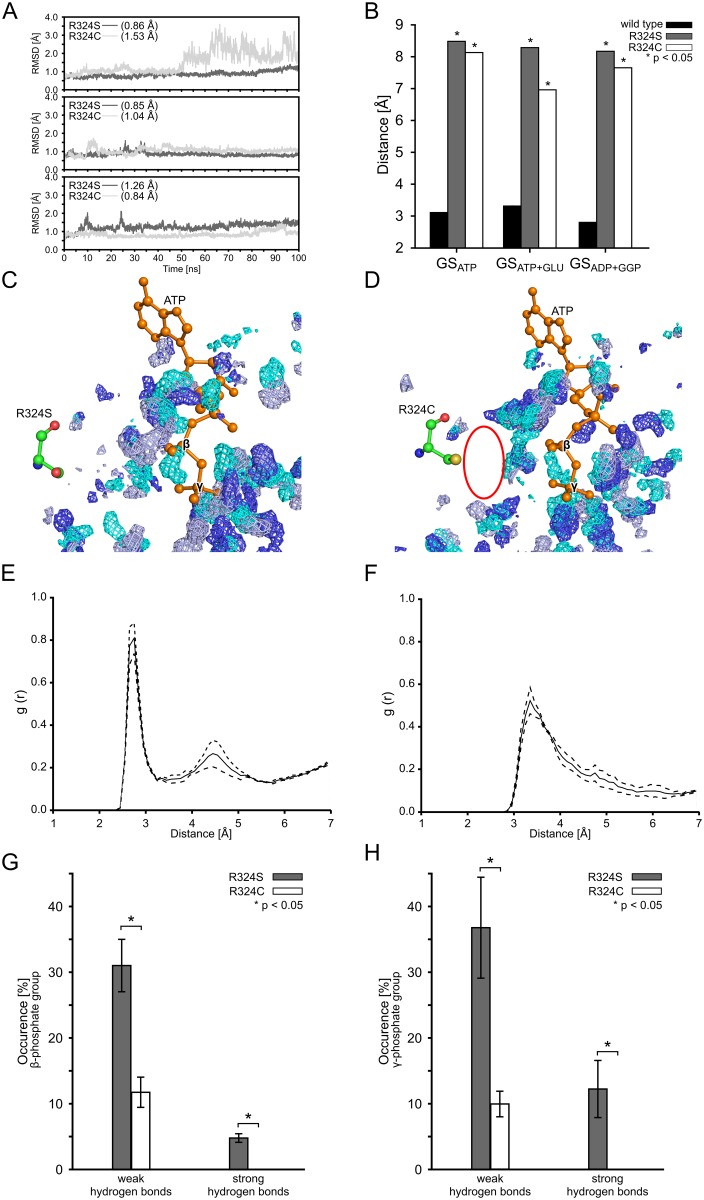
Structural changes and water structure in the binding sites of the R324S and R324C mutants. **(A):** Backbone RMSD of catalytic site residues (for definition see Fig 1E) of the R324S (dark grey) and R324C (light grey) mutants in the GS_APO_ state during 100 ns of MD simulations (each subpanel shows MD simulations initiated from a different starting structure (see section “Experimental procedures” above)). Respective mean RMSD values are listed in brackets; SEM < 0.1 Å in all cases. (**B):** Mean distances between R324 (wild type GS), or S324 and C324 in GS mutants, and the β-phosphate group of ATP in states GS_ATP_ and GS_ATP+GLU_ or ADP in state GS_ADP+GGP_, respectively. Stars indicate significant differences (*p* < 0.05) with respect to the wild type. In all cases, SEM < 0.1 Å. (**C, D):** Density distribution of water around ATP in the binding site during MD simulations of R324S (C) and R324C (D) in the GS_ATP+GLU_ state. Regions where water is most present are indicated by water density grids for three MD simulations (cyan, light blue, and dark blue; isopleths were plotted such that they encompass 80% of the maximum occupancy). ATP (orange) and the mutated amino acid 324 are shown in ball-and-stick representation. The red oval indicates an area of pronounced difference in the water density between the R324S and R342C mutants. (**E, F):** Radial distribution function (RDF) of water oxygens around the side chain oxygen or sulfur, respectively, of S324 (E) and C324 (F) in the GS_ATP+GLU_ state. The solid line shows the mean RDF, and dashed lines indicate ± SEM. (**G, H):** Mean relative occurrence of water-mediated hydrogen bonds between the β-phosphate group (G) or the γ-phosphate group (H) of ATP and residues S324 (gray) or C324 (white), respectively, in the GS_ATP+GLU_ state. The distance cutoff for the hydrogen bonds was set to 2.8 Å for strong hydrogen bonds and 3.2 Å for weak hydrogen bonds. Error bars denote the SEM; stars indicate a significant difference (*p* < 0.05) between both mutants. For panels B—H, data from the 20–100 ns intervals of the respective MD simulations was taken.

Residue R324 is located in the catalytic site and forms a salt bridge with the β-phosphate group of ADP (Fig 1C) [22]. Although no crystal structure information for ATP-bound human GS is available, it is likely that R324 is interacting with the β-phosphate group of ATP, too. The substitution of R324 with cysteine reduces GS activity to about 12% of that of the wild type [17]. The substitution of R324 with serine likely partially conserves GS activity [20]. Initially, we investigated whether the R324S or R324C mutations induce structural changes within the catalytic site. For this, we computed the backbone RMSD of the residues of the catalytic site (Fig 1E) for both mutants in the GS_APO_ state (Fig 2A). In the case of the R324S mutant, the RMSD remains largely constant during three independent MD simulations (mean RMSD: 0.86 Å, 0.85 Å, and 1.26 Å (SEM < 0.1 Å)) (Fig 2A). In the case of the R324C mutant, the RMSD remains largely constant in two MD simulations (mean RMSD: 1.04 Å and 0.84 Å (SEM < 0.1 Å)) (Fig 2A). During one MD simulation, however, the RMSD fluctuates up to 3.5 Å, with a mean RMSD of 1.53 Å (SEM < 0.1 Å) (Fig 2A). Visual inspection of the respective trajectory revealed a highly mobile loop (termed “Glu flap” [21, 24]), formed by residues 304–306, as the cause for these fluctuations; excluding those three residues results in a mean RMSD of 0.95 Å (SEM < 0.1). All mean RMSD values are thus comparable with the mean RMSD obtained for the catalytic site of wild type GS (Fig1F). This demonstrates that neither the R324S nor the R324C mutation changes the catalytic site structure markedly.

Next, we hypothesized that differences in GS activity in the R324C and R324S mutants arise from differences in the interactions between arginine and the mutated residues, respectively, with ATP in the first step of glutamine formation. To investigate this, we subjected wild type GS and the R324C and R324S mutants in the GS_ATP_, GS_ATP+GLU_, and GS_ADP+GGP_ states to MD simulations. Distances were measured over the respective structural ensembles between the terminal guanidine nitrogens of R324 and oxygens oriented towards R324 of the β-phosphate group of ADP in the GS_ADP+GGP_ state, or ATP in the GS_ATP_ and GS_ATP+GLU_ states. The distance measurements confirmed the existence of a salt bridge interaction in the wild type for ADP [22] and revealed such an interaction for ATP (mean distances < 3.5 Å (SEM < 0.1 Å)) (Fig 2B), which is lower than the threshold of 4 Å used to define a salt bridge interaction [66]. This interaction is permanently present in eight out of nine trajectories (Figure A in S1 file), and is formed after ~65 ns in one trajectory (Figure A in S1 file) and remains stable thereafter. In contrast, in both R324 mutants, the mean distances between the thiol group of cysteine and the hydroxyl group of serine, respectively, and the β-phosphate group of ADP or ATP are > 7 Å (SEM < 0.1 Å) (Fig 2B). The differences in the mean distances with respect to the wild type are significant (*p* < 0.05). In addition, time series of the distances over the course of the MD simulations did not show the formation of hydrogen bonds between S324 or C324 and the β-phosphate group of ADP or ATP, respectively, in any conformation (Figure A in S1 file). Thus, neither residue at position 324 forms a direct hydrogen bond with the β-phosphate group of ADP or ATP in the R324C and R324S mutants. The lack of a direct interaction between the residue at position 324 and the β-phosphate group of ATP could lead to an increased mobility of ATP within the catalytic site. This could distort the proper mutual arrangement of the substrates prior to the reaction, leading to a loss in catalytic activity. However, the RMSF of ATP bound to wild type GS or one of the mutants do not differ significantly (Figure B in S1 file), which suggests that the mutation at position 324 may rather influence the affinity of the substrate ATP towards GS (see section “Relative effective binding energies of GS substrates” below).

Subsequently, we investigated why the R324S mutant likely leads to a higher residual GS activity than the R324C mutant [20]. We hypothesized that the direct interaction between the sidechain of R324 in the wild type could be replaced by water-mediated interactions in the R324S mutant but not the R324C mutant. Therefore, we determined the water density between residue 324 and ATP in MD trajectories by counting the presence of water molecules within a cubic grid of 0.33 Å spacing. Isopleth plots of the density distribution that encompass 80% of the maximum occupancy of water molecules in three independent MD simulations of the R324S and R324C mutants, respectively, in the GS_ATP+GLU_ state are shown in Fig 2C and 2D. These results qualitatively reveal a much broader region of high water density between R324S and the β-and γ-phosphate groups of ATP than in the R324C case, resulting in regions of high water occupancy close to the R324S side chain. For a quantitative analysis, radial distribution functions (RDF) for oxygen atoms of water molecules around the side chain oxygen (R324S) or sulfur (R324C) were computed (Fig 2E and 2F). The RDF reveals two shells of water molecules around R324S, with the first shell peaking at ~2.8 Å, consistent with previous findings [67], and the second shell at ~4.5 Å (Fig 2E). The distance of the first shell peak is in line with that of a strong hydrogen bond (2.5–3.2 Å) [48]. In the case of R324C, the first shell peaks at ~3.2 Å (Fig 2F). This difference with respect to R324S reflects a similar difference of the van der Waals radii of oxygen and sulfur [68]. More notable, the water density at the position of the first shell is 30% higher in the case of R324S than for R324C, and the second shell is considerably more structured in the former case (Fig 2E), demonstrating stronger hydrogen bonding interactions between serine and water, as expected [69].

Finally, we determined the frequency of occurrence of water-mediated hydrogen bonds between R324S or R324C and the β- and γ-phosphate groups of ATP (Fig 2G and 2H). From the distance between the two side chains and the β-phosphate group of ∼7.0–8.5 Å, respectively, (Fig 2B) and a water diameter of 2.9 Å [70], one can deduce that between two to three water molecules can bridge this gap. In the analysis, we distinguished between weak (distance cutoff between hydrogen bond donor and acceptor of *d*_*cut*_ = 3.2 Å) and strong (*d*_*cut*_ = 2.8 Å) hydrogen bonds. Only hydrogen bonds with a distance < *d*_*cut*_ were considered for analyses. The frequency of occurrence of water-mediated hydrogen bonds between R324S and the β- and γ-phosphate groups of ATP is significantly higher than for R324C for both weak (31.0 ± 4.0% *versus* 11.8 ± 2.3%; 36.8 ± 7.7% *versus* 9.9 ± 2.0%; Fig 2G and 2H) and strong (4.8 ± 0.7% *versus* 0%; 12.2 ± 4.4% *versus* 0%; Fig 2G and 2H) hydrogen bonds.

In summary, our analyses show the absence of a direct hydrogen bonding interaction between the side chains of R324S and R324C mutants and the β-phosphate group of ATP, in contrast to the wild type GS. In the case of the R324S mutant, the direct interaction is replaced by water-mediated hydrogen bonds; such hydrogen bonds are significantly less frequently observed in the R324C mutant.

### Effect of the R341C mutation on the first step of the catalytic cycle

**Fig 3:**
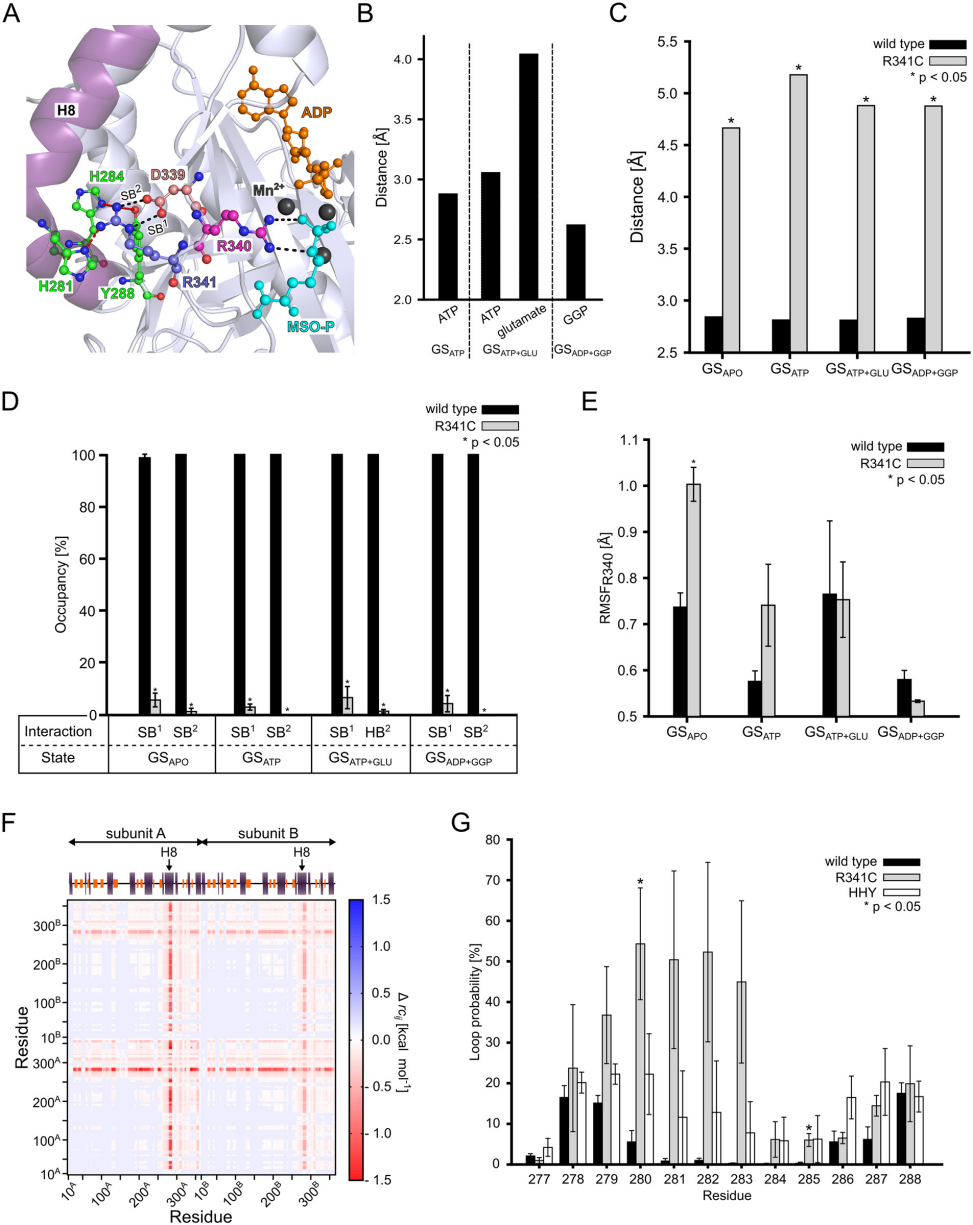
Structural and stability changes in the R341C mutant. **(A):** Close-up view of the crystal structure of human GS (PDB entry 2QC8 [22]) around R341. The triad composed of residues D339, R340, and R341, and residues H281, H284, and Y288 on helix 8 (H8; raspberry) are shown in ball-and-stick representation. The salt bridge between D339 and R341 (SB^1^ and SB^2^)) and the interaction between R340 and L-methionine-*S*-sulfoximine phosphate (MSO-P) are indicated by black dashed lines. Interactions between R341 and H281, H284, and Y288, respectively, are indicated by red dashed lines. ADP (orange) and MSO-P (cyan) are depicted in ball-and-stick representation, and Mn^2+^ ions are shown as black spheres. (**B):** Mean distances between terminal guanidino nitrogens in R340 and the oxygens of the γ-phosphate group of ATP oriented towards R340, the center of oxygens of the γ-carboxylic group of glutamate, and the carbonylic oxygen in GGP. SEM < 0.1 Å in all cases. GS_ATP_, GS_ATP+GLU_, and GS_ADP+GGP_ were considered. (**C):** Mean distances of interactions SB^1^ and SB^2^ (see panel A) for wild type GS and when considering the thiol group of C341 in the R341C mutant. SEM < 0.1 Å in all cases. Stars indicate a significant difference (*p* < 0.05) between wild type and mutant. (**D):** Mean occupancy of interactions SB^1^ and SB^2^ (see panel A) for wild type GS and when considering the thiol group of C341 in the R341C mutant. Error bars denote the SEM; stars indicate a significant difference (*p* < 0.05) between wild type and mutant. **(E):** All-atom RMSF of residue R340 in wild type GS and the R341C mutant. Error bars denote the SEM; stars indicate a significant difference (*p* < 0.05) between wild type and mutant. (**F):** Stability map depicting significant differences (*p* < 0.05) in the structural stability as computed by CNA between wild type GS and the R341A mutant. Protein structures were extracted from the GS_ADP+GGP_ state: Blue colors indicate that two residues are less stably connected in wild type, red colors that two residues are less stably connected in the R341A mutant. The secondary structure of GS is depicted on the top, with orange bars representing β-strands and blue bars representing α-helices; H8 is labelled. Subunits are indicated by arrows. (**G):** Probability for residues 277 to 288 of H8 to be in a loop conformation during MD simulations of wild type GS, the R341C mutant, and the HHY mutant in the GS_ATP_ state. Error bars denote the SEM; stars indicate significant differences (*p* < 0.05) with respect to the wild type. Results in panels B-G are based on snapshots recorded during the 20–100 ns interval of the respective MD simulations.

In fibroblasts from the father of a patient with an R341C mutation in GS, a 50% drop in specific GS activity was found [17]. As R341 is located at a distance of 10 Å from the catalytic site (Fig 1C) and its side chain points away from this site (Fig 3A), the influence of the R341C mutation on GS activity must arise from a long-range effect that percolates through the GS structure. Analysis of the GS crystal structure [22] revealed R341 as part of an amino acid triplet (Fig 3A; termed triad hereafter) consisting of R341, D339, and R340. While R341 is at the most distant end of the triad with respect to the catalytic site, R340 makes hydrogen bond and salt bridge interactions with the sulfoximine phosphate part of MSO-P (Fig 3A).

During our MD simulations of wild type GS in the GS_ADP+GGP_ state, we also observe a hydrogen bond between R340 and GGP (mean distance between the terminal guanidine nitrogens of R340 and the carbonyl oxygen in GGP: 2.63 Å (SEM < 0.1 Å); Fig 3B), in agreement with the crystal structure. This interaction is stable over 100 ns MD simulations (Figure C in S1 file). R340 does not interact with glutamate in the GS_ATP+GLU_ state, however (mean distance between the center of the terminal guanidine nitrogens of R340 and the center of the γ-carboxylic function in glutamate: 4.05 Å (SEM < 0.1 Å); Fig 3B, Figure C in S1 file). Rather, R340 interacts with the γ-phosphate group of ATP in the GS_ATP+GLU_ state (mean distance between the center of the terminal arginine nitrogens and the center of the oxygens oriented towards R340 of the γ-phosphate group of ATP: 3.06 Å (SEM < 0.1 Å)) as it does in the GS_ATP_ state (mean distance between the center of the terminal arginine nitrogens and the center of the oxygens oriented towards R340 of the γ-phosphate group of ATP: 2.88 Å (SEM < 0.1 Å)) (Fig 3B). Again, the interactions with the γ-phosphate group are stable over the course of 100 ns MD simulations (Figure C in S1 file). The observed shift of the interaction of R340 with ATP in the GS_ATP_ and GS_ATP+GLU_ states to one with GGP in the GS_ADP+GGP_ state suggests a prominent involvement of R340 in the first step of the catalytic cycle of GS.

Within the triad, D339 and R341 form a salt bridge in the crystal structure (Fig 3A, [22]). The mean distances SB^1^ and SB^2^ (Fig 3A, black dashed lines) between side chains of D339 and R341 are < 3 Å (SEM < 0.1 Å) in all states for wild type GS (Fig 3C). This interaction is present ≥ 98% of the time during MD simulations of wild type GS of all states (Fig 3D, SB^1^ and SB^2^ occupancy determined for salt bridges with *d*_*cut*_ < 4.0 Å). These findings suggest that D339 and R341 stabilize R340 as flanking residues. This stabilization counteracts the inherent flexibility of the loop region all three residues are located in (Fig 3A). In the R341C mutant, the mean distances SB^1^ and SB^2^ between the carboxylate oxygens of D339 and the thiol function of C341 is > 4.5 Å (SEM < 0.1) in all states (Fig 3C). Employing *d*_*cut*_ < 3.2 Å and angle < 120°, a hydrogen bond between the carboxylate oxygens of D339 and the thiol function of C341 is present in ≤ 5% of the time during MD simulations of all states (Fig 3D). These results suggest that the stabilizing effect of residue 341 on R340 is largely lost in the R341C mutation. To substantiate this, we computed residue-wise RMSF of R340 for wild type GS and the R341C mutant in all states (Fig 3E). For GS_APO_, the RMSF is significantly (*p* < 0.05) larger in the R341C mutant (1.00 ± 0.03 Å) than the wild type GS (0.74 ± 0.03 Å) (Fig 3E). In contrast, no significant changes were observed for states GS_ATP_, GS_ATP+GLU_, and GS_ADP+GGP_ (Fig 3E), likely because R340 is then stabilized by interactions with the substrates (see above; Fig 3B). These results suggest that the R341C mutation indirectly affects the first step of the catalytic cycle, particularly ATP binding, by influencing the R340 mobility in the GS_APO_ state.

### Effect of the R341C mutation on signal transmission between the catalytic site and helix 8

In order to investigate the influence of mutant R341C on GS’ mechanical stability, we applied Constrained Network Analysis (CNA), where biomolecular structures are represented as molecular frameworks [26] and analyzed by means of rigidity theory [71]. That way, regions that are either structurally stable (“rigid”) or flexible are identified. We compared results for wild type GS to those obtained for a perturbed structural ensemble in which interactions by the side chain of R341 are abolished (see “Experimental procedures” for how this ensemble was generated). The result is depicted as a difference stability map Δ*rc*_*ij*_ [51], which shows if a rigid contact between two residues becomes less stable in the perturbed structural ensemble (red colors in Fig 3F; a rigid contact exists if two residues belong to the same rigid region). The loss of side chain interactions of R341 results in a significant (*p* < 0.05) destabilization of the C-terminus of the GS dimer model (residues 265–365; Fig 3F). Control calculations performed for the GS decamer corroborate this finding (Figure D in S1 file). In this region, helix 8 (H8, residues 266–288), located on the outside of the GS subunit (Fig 3A), shows the most pronounced loss in structural stability (with Δ*rc*_ij_ values of -1.4 kcal·mol^-1^; see ref. [25] for an explanation of the energy values). Visual inspection of the GS crystal structure [22] reveals that the guanidino group of R341 forms hydrogen bonds with three residues on H8 (H281, H284, and Y288) (Fig 3A).

To substantiate these results, we analyzed the structural stability of H8 during MD simulations of wild type GS and the R341C mutant in the GS_ATP_ state, as well as of an H281A/H284A/Y288A triple mutant in the same state (hereafter termed HHY mutant). The HHY mutant serves as a mimic of the R341C mutant because here, as in the R341C mutant, no hydrogen bonds between residue 341 and residues 281, 284, and 288, respectively, can be formed. We computed the probability that residues 266–288 form a loop in the course of the MD simulations (Fig 3G). For wild type GS, this loop probability is below 20% for residues 278, 279, and 288, below 10% for residues 280, 286, and 287, and below 5% for the remaining amino acids (Fig 3G). For the R341C mutant, in contrast, a markedly increased loop probability is found for residues 279 to 283 (up to 50%; Fig 3G). In this region, mutant HHY shows the most distinct increases in the loop probability compared to wild type GS, too (up to 22%; Fig 3G). For residues 266–276, there are no differences in loop probability between wild type GS and R341C or HHY mutant, respectively. These results demonstrate that R341 has a stabilizing influence on H8 in wild type GS; this influence is lost in both the R341C and HHY mutants. Considering the above results on R341’s role in the triad, this suggests that R341 can relay information between the catalytic site and H8. Of note in this context, Krajewski *et al*. observed a shift of H8 in the first step of the catalytic cycle that closes GS’ catalytic site when ATP is bound [22]; this ATP binding-induced shift has been recognized as a prerequisite for glutamate binding [22]. Hence, we hypothesized that the loss of the relaying function in the R341C mutant hampers glutamate binding to GS. For the same reason, glutamate binding should be hampered in the HHY mutant.

### Overexpression of glutamine synthetase in human embryonic kidney cells

**Fig 4:**
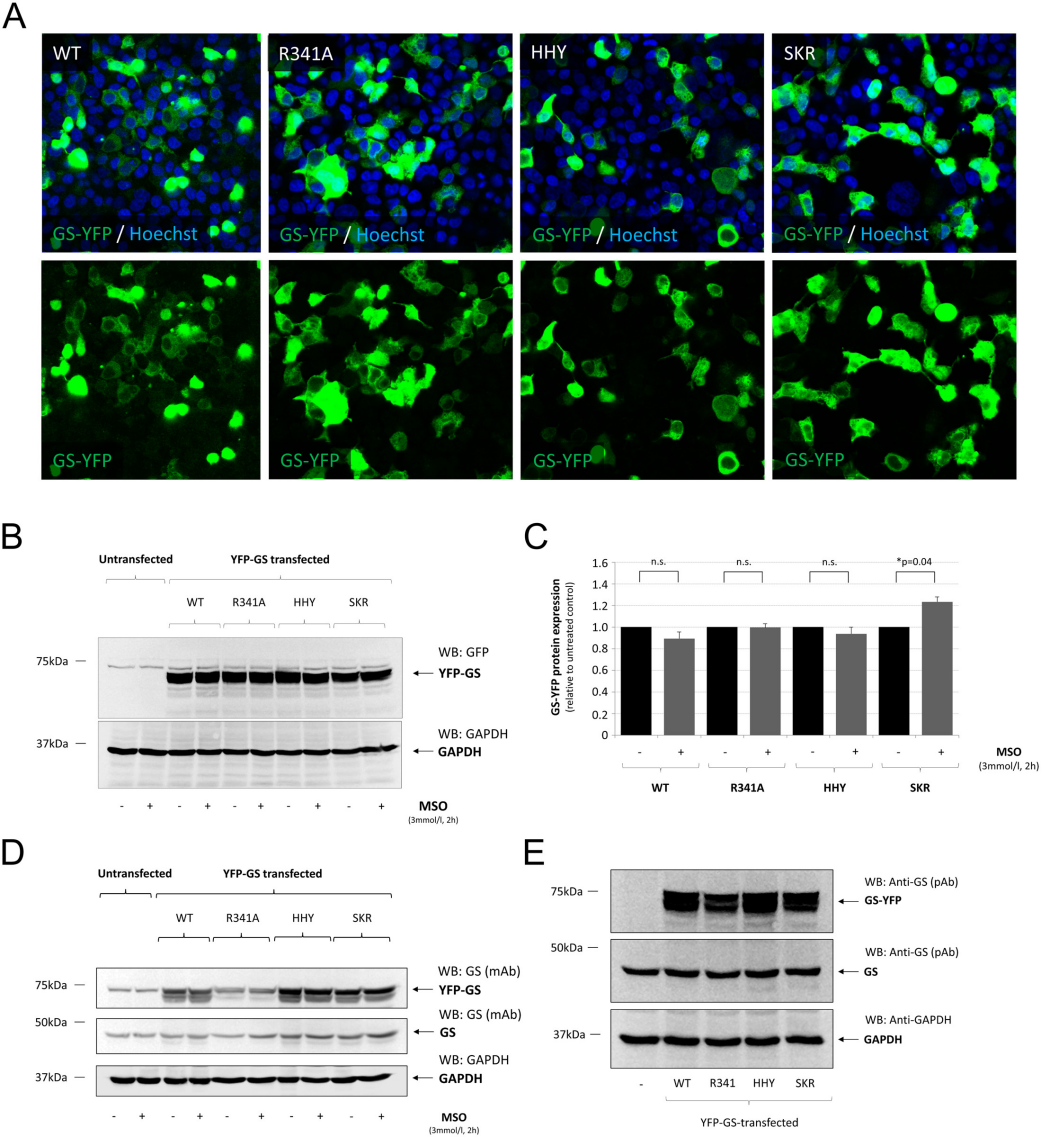
Verification of GS-YFP transfection and overexpression in human embryonic kidney cells. Human embryonic kidney cells (HEK293) were transfected with cDNA constructs coding for YFP-tagged human glutamine synthetase (GS-YFP) without (WT) or with mutations at the indicated positions within the GS amino acid sequence (H281A, H284A, Y288A = HHY; S278A, K279A, R280A = SKR). HEK293 cells were either left untreated or were treated with the GS-inhibitor L-methionine-*S*-sulfoximine (MSO, 3 mmol/l, 2 h). **(A):** Transfection efficiency was verified by confocal laserscanning microscopy in Hoechst34580 stained cells. (**B):** Detection of YFP-GS by Western-blot using anti-GFP antibodies. (**C):** Densitometric quantification of GS-YFP expression levels as detected by anti-GFP antibodies. Anti-GFP immunoreactivity in MSO-treated cells is given relative to the respective untreated control. (**D):** Detection of GS by Western-blot after heat- and detergent-mediated release of MSO from the enzyme, which restores recognition of GS by the monoclonal anti-GS antibody (BD, clone6). GAPDH served as a loading control. **(E):** Detection of overexpressed human GS-YFP and endogenously expressed GS by Western-blot using polyclonal anti-GS antibodies (Sigma, Deisenhofen, Germany). GAPDH served as a loading control. * Statisticially significantly different compared to untreated YFP-GS-transfected HEK293 cells (*p* < 0.05). n.s.: not statistically significantly different.

YFP-tagged wild type and mutated (R341A; HHY; S278A/K279A/R280A = SKR, the latter mutant was introduced as a negative control to HHY, as S278, K279, and R280 are located on H8 but do not interact with R341) human GS were transiently expressed in HEK293 cells. Expression of human GS-YFP in HEK293 cells was monitored by confocal laser scanning microscopy (Fig 4A) and verified by Western-blot analysis, which showed that YFP-tagged GS-constructs coding for human GS were strongly expressed in HEK293 cells (Fig 4B). Treating GS-YFP transfected HEK293 cells with MSO (3 mmol/l, 2 h) had no effect on GS-YFP expression levels in wild type-, R341A-, or HHY-treated cells compared to the respective control (Fig 4B and 4C). However, anti-YFP-immunoreactivity was slightly elevated in MSO-treated HEK293 cells transfected with SKR-mutated GS-YFP (Fig 4B and 4C).

GS was also detected in GS-YFP transfected cells by Western-blot using a monoclonal antibody formerly shown not to react with GS when arginine at position 341 was mutated to cysteine (R341C) (15). As shown in Fig 4D, the monoclonal anti-GS antibody strongly detected overexpressed wild type-GS as well as HHY- and SKR-mutated GS-YFP but failed to detect GS when arginine 341 is mutated to alanine (R341A). In contrast, when using a polyclonal antibody (Sigma, Deisenhofen, Germany) raised against the C-terminus of GS (amino acids 357–373), GS-YFP mutated on arginine 341 (R341A) was readily detected, as were wildtype- and HHY- or SKR-mutated GS-YFP (Fig 4E). MSO-treatment had no effect on detectability of GS-YFP by Western-blot using the monoclonal antibody raised against GS (Fig 4D).

HEK293 cells also expressed GS endogenously (Fig 4D and 4E). However, overexpression of GS-YFP (Fig 4D and 4E) as well as MSO-treatment in GS-YFP transfected HEK293 cells had no effect on endogeneous GS expression levels (Fig 4D).

The results show that wildtype-, as well as R341A-, HHY- and SKR-mutated GS-YFP is efficiently expressed in HEK293 cells by lipofection and that GS-YFP expression levels are not affected by MSO-treatment compared to the respective control. The results also show that wildtype-, HHY- and SKR-mutated but not R341A-mutated GS is recognized by a monoclonal antibody raised against GS (BD, clone 6) in Western-blot. This is explained by the specificity of a monoclonal antibody, which recognizes only a single epitope, and by the interaction of the paratope of an antibody with an epitope, which is mediated by only about 5 amino acids [72]. Therefore, mutations that are located far away from the presumed recognition site (R341) such as S278 are not expected to impair the binding of the anti-GS antibody.

### Effect of mutating amino acids H281, H284 and Y288 or S278, K279 and R280 on binding of L-methionine-*S*-sulfoximine to glutamine synthetase

MSO binds non-covalently to the glutamate-binding pocket of GS [73] thereby masking an epitop which is recognized by a monoclonal antibody (BD, clone 6) [62]. Therefore, loss of anti-GS immunoreactivity after MSO treatment may serve as a surrogate marker for glutamate binding to the catalytic site of GS.

**Fig 5:**
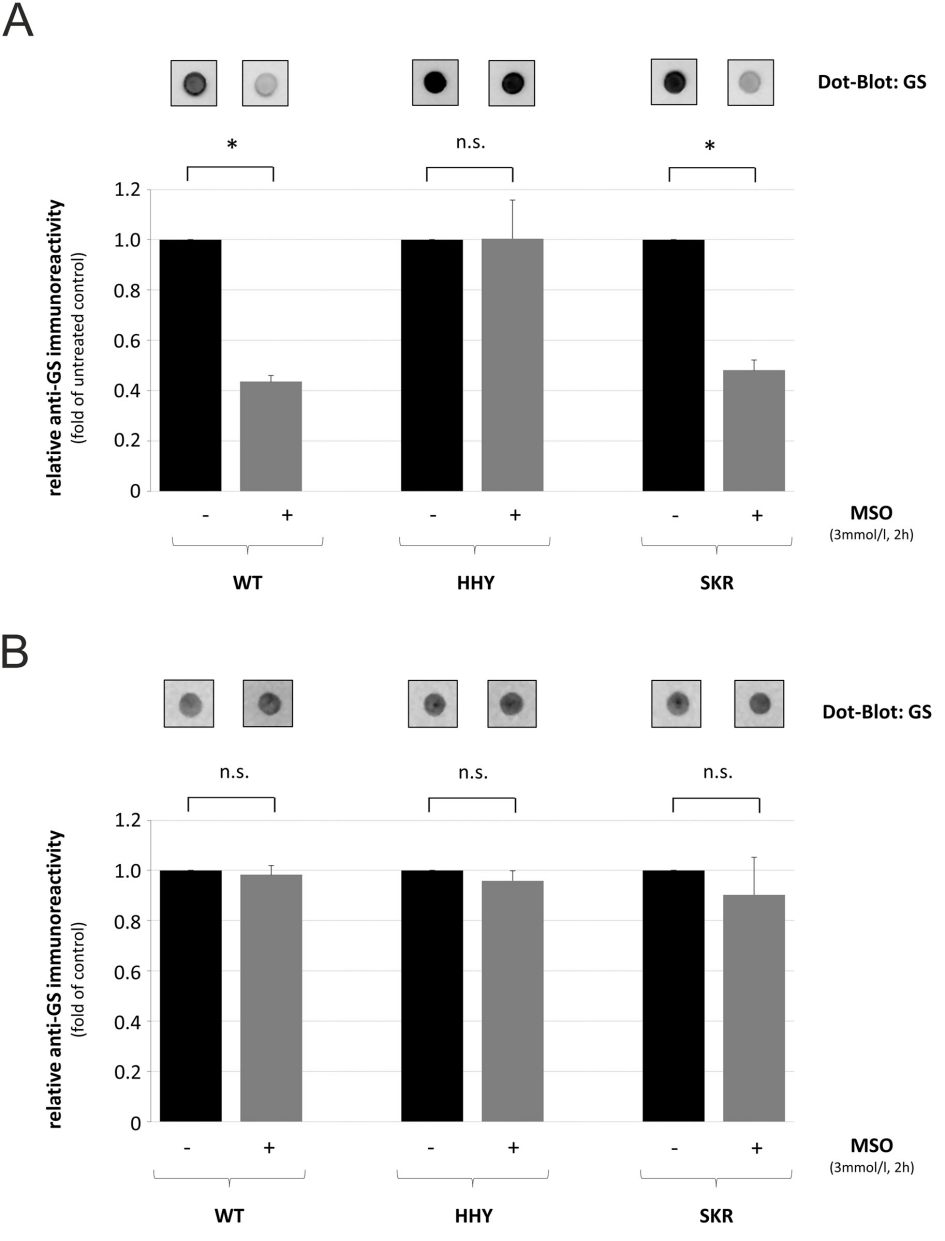
Detection of GS by dot-blot analysis in human embryonic kidney cells. Glutamine synthetase-YFP-transfected human embryonic kidney cells (HEK293) were either left untreated or treated with the GS-inhibitor L-methionine-*S*-sulfoximine (MSO) for 2 h. Equal amounts of **(A)** native or **(B)** heat- and detergent-denaturated protein were spotted onto a nitrocellulose membrane, and GS was detected by dot-blot analysis followed by densitometric quantification of anti-GS immunoreactivity. Anti-GS immunoreactivity in MSO-treated cells is given relative to the respective untreated control. * Statisticially significantly different compared to untreated controls (*p* < 0.05). n.s.: not statistically significantly different.

As shown by dot-blot analysis using native protein, anti-GS immunoreactivity was significantly diminished by about 50% after MSO-treatment in HEK293 cells transfected with wild type- or SKR-mutated GS but remained unchanged in HEK293 cells expressing HHY-mutated GS compared to untreated controls (Fig 5A). In contrast, upon heat- and detergent-mediated protein denaturation, anti-GS immunoreactivity was similar in untreated and MSO-treated, GS-transfected HEK293 cells (Fig 5B).

The results suggest that MSO binds to the glutamate-binding pocket of wildtype- and SKR-mutated-GS but not to the pocket of HHY-mutated GS.

### Relative effective binding energies of GS substrates

**Fig 6:**
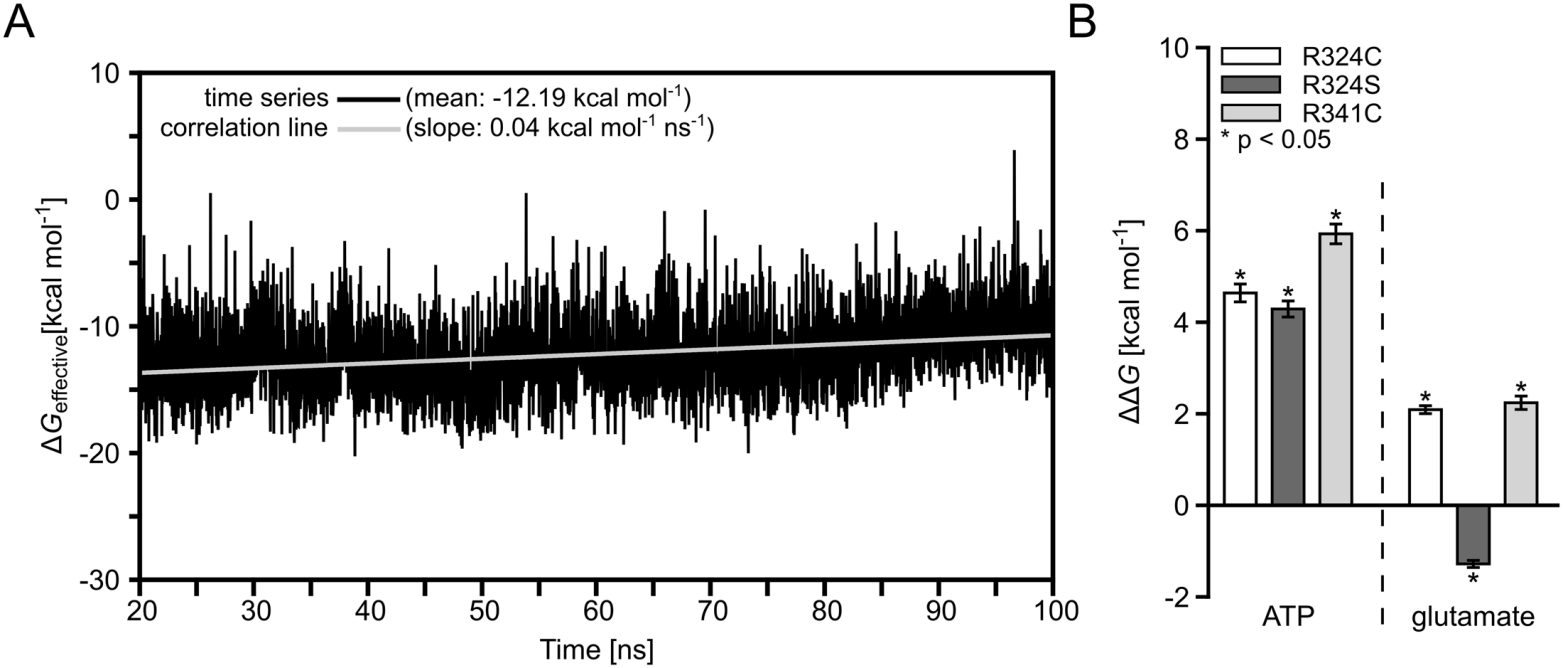
Mean relative effective binding energies of ATP or glutamate. **(A):** Time-series of effective binding energies calculated for 4000 snapshots extracted in 20 ps intervals from the last 80 ns of MD simulations of glutamate bound to wild type GS in the GS_ATP+GLU_ state (black line) and least-squares line of best fit from a correlation analysis (grey line). The mean of the effective binding energies and the slope of the least-squares line of best fit are given in the legend. (**B):** Mean effective binding energies with respect to wild type GS (ΔΔ*G*, eq 1). ΔΔ*G* values were calculated by the MM-PBSA approach for ATP in the GS_ATP_ state and for glutamate in the GS_ATP+GLU_ state for GS mutants R324C, R324S, and R341C. Error bars indicate *SEM*_total_ (eq 3); stars indicate a significant difference (*p* < 0.05) between wild type and mutant.

In order to determine energetic consequences of the GS mutations on substrate binding, effective binding energies relative to wild type GS (ΔΔ*G*, eq 1) were computed by the MM-PBSA approach for ATP bound to the R342S, R342C, and R341C mutants in the GS_ATP_ state and for glutamate bound to R342S, R342C, and R341C in the GS_ATP+GLU_ state. The average drift in the effective binding energy Δ*G*_wildtype_ of glutamate binding to the GS_ATP+GLU_ state over the last 80 ns of the MD simulations used for analysis is 0.04 kcal·mol^-1^·ns^-1^, as determined by the slope of the least-squares line of best fit from a correlation analysis (Fig 6A). The magnitude of the average drifts in all other effective binding energies Δ*G*_wildtype_ or Δ*G*_mutant_ is < 0.15 kcal mol^-1^ ns^-1^ (Figure E and F in S1 file). The magnitude of these drifts is comparable to those found for ligands binding to other proteins [74] or ribosomal RNA [64] and indicates converged estimates of Δ*G*_wild type_ or Δ*G*_mutant_.

The computed ΔΔ*G* for ATP in the GS_ATP_ state is 4.29 ± 0.19 kcal·mol^-1^ for the R324S mutant and 4.64 ± 0.21 kcal·mol^-1^ for the R324C mutant (Fig 6B). While these results demonstrate that ATP binding to the mutants is disfavorable compared to binding to wild type GS, the difference between the two mutants is not significant. The latter finding is unexpected considering that both mutations lead to different clinical outcomes. Likely, the similar ΔΔ*G* result from neglecting explicit water molecules in the MM-PBSA computations, which, consequently, results in missing favorable energetic contributions due to water-mediated hydrogen bonds between S324 and ATP, as observed in the MD simulations (Fig 2G and 2H). Including tightly bound structural water molecules in MM-PBSA calculations may provide a possibility to overcome this shortcoming [55, 75]. However, in the case of R324S GS, this approach does not appear applicable to us as we observed frequent exchanges of water molecules involved in the hydrogen bond formations.

For mutant R341C, ΔΔ*G* is 5.93 ± 0.23 kcal·mol^-1^ for ATP and 2.24 ± 0.16 kcal·mol^-1^ for glutamate binding (Fig 6B). Thus, our results show that ATP binding is weakened in all GS mutants; at *T* = 300 K, the magnitudes of ΔΔ*G* relate to decreases in the binding constants of ATP of 1.6 to 4.3 log units. From a qualitative point of view, the weakening in the R341C mutant is in line with the above findings that the mutation results in a destabilization of R340 (Fig 3E), which, in turn, interacts with ATP in the GS_ATP_ state (Fig 3B, Figure C in S1 file). From a quantitative point of view, it is surprising that of all mutants the R341C mutant shows the largest effect on ATP binding, although residue 341 does not directly interact with ATP. Part of this effect may be caused by neglecting energetic contributions due to conformational changes in the solutes upon binding in the 1-trajectory approach pursued here, or by neglecting changes in the configurational entropy of the solutes upon complex formation (see section Materials and Methods).

Glutamate binding is weakened in the R324C (R341C) GS mutant by ΔΔ*G =* 2.09 ± 0.10 kcal mol^-1^ (ΔΔ*G =* 2.29 ± 0.16 kcal mol^-1^) but not in R324S GS (ΔΔ*G* = -1.28 ± 0.09 kcal mol^-1^) (Fig 6B). Considering that, according to Krajewski *et al*. [22], an ATP binding-induced conformational change in GS’ catalytic site is a prerequisite for glutamate binding, our findings are in line with our above structural and energetic analyses according to which ATP binding is particularly weakened in the R324C and R341C mutants.

## Discussion

The molecular mechanisms of how the three mutations R324C, R324S, and R341C in human GS [17, 19] lead to a glutamine deficiency, resulting in neonatal death in the case of R324C and R341C, have not been understood. Furthermore, it has remained elusive why the R324S mutation, but not the R324C mutation, likely partially conserves GS activity [20]. Here, we show by MD simulations and binding free energy calculations that both R324 mutations lead to a loss of direct interactions with the β-phosphate group of ADP or ATP compared to wild type GS, and weakened ATP binding. In the case of the R324S mutant, the direct interaction is replaced by water-mediated hydrogen bonds, which are significantly less frequently observed in the R324C mutant. MD simulations and binding free energy calculations demonstrate that the R341C mutation indirectly weakens ATP binding. In addition, rigidity analysis reveals that the R341C mutation particularly destabilizes helix H8, which should hamper glutamate binding to GS; *in vitro* studies provide evidence for this influence.

Initially, we established a model system consisting of two adjacent subunits of the GS decamer forming a single catalytic site for performing MD simulations in a computationally efficient way. During 100 ns of MD simulations, small structural deviations (RMSD < 1.7 Å; Fig 1D) from the starting structure were found for the core regions of both the dimeric model and the decamer reference. Likewise, structural deviations of the binding site region and the bound ligands computed for the dimeric model were at the lower end (RMSD = 0.89 Å (Fig 1F), 0.49 Å (Fig 1G), and 0.96 Å (Fig 1H), respectively) of the range of values found for the decamer (RMSD < 2.3 Å (Fig 1F) and < 0.7 Å (Fig 1G) or < 0.7 Å (Fig 1H), respectively). Thus, no gross conformational changes were observed for the dimeric model despite the lack of interactions to neighboring subunits. These results are in agreement with a crystallographic study that shows no major allosteric changes within or between the pentamers of human GS upon MSO binding as well as only small (RMSD < 0.35 Å) structural differences between canine GS in the *apo* state and human GS in ligand-bound states [22]. Similarly, only small structural alterations in catalytic site loops during catalysis have been reported for prokaryotic GS of class I-β [21, 24]. Regarding the divalent metal ions required by eukaryotic GS for activity [35], we considered Mg^2+^ ions in our dimeric model system rather than Mn^2+^ ions. We did so as only 20–30% of GS subunits from ovine brain tissue have been found trapped with Mn^2+^ under physiological Mg^2+^ and Mn^2+^ concentrations [76], and human GS from brain is 10-fold more active with Mg^2+^ bound than with Mn^2+^ [77]. For investigating the effects of the R324S, R324C, and R314C mutations, three independent MD simulations were performed for each of the four states of GS (GS_APO_, GS_ATP_, GS_ATP+GLU_, GS_ADP+GGP_) of wild type GS and the three mutants, respectively. The replicate MD simulations allow probing for the influence of the starting conditions and determining the statistical significance of the computed results [78].

Regarding the R324S and R324C mutations, we first tested if they distort the structure of the catalytic site in the GS_APO_ state. We excluded residues of the Glu flap loop (residues 304–306) from the analysis because this loop was identified to be highly mobile in a previous study [24], which is in line with our results (Fig 1E). For both mutants, the mean backbone RMSD of the residues of the catalytic site is ≤ 1.26 Å with respect to the starting structure (Fig 2A). These conformational changes are only slightly larger than those found in the MD simulations of the dimeric model of wild type GS (RMSD = 0.89 Å (Fig 1F)), which demonstrates that neither mutation changes the catalytic site markedly. In contrast, a significant difference between the wild type residue R324 and serine or cysteine at position 324 is found with respect to interactions with the β-phosphate group of bound ADP or ATP: R324 forms salt bridge interactions with the substrates, respectively (Fig 2B), as also observed in the crystal structure of human GS complexed with ADP [22]; however, neither do S324 nor C324 interact directly with ADP or ATP (Fig 2B). This interaction loss does not result in a significant change of the ATP mobility within the catalytic site of the R324S and R324C mutants compared to wild type GS, likely reflecting the structure-stabilizing influence of the Mg^2+^ ion at the metal binding site n2, which interacts with the nucleotide [24, 79]. Yet, the mutations result in effective energies for binding of ATP that are disfavorable by about 4 kcal mol^-1^ with respect to wild type GS (Fig 6B). Neglecting contributions due to changes in the configurational entropy of the solute molecules upon binding [53, 59, 61], this change in the effective binding energies is equivalent to an about 1000-fold lower association constant of ATP. Taken together, these results strongly suggest that the R324S and R324C mutations deteriorate GS catalytic activity by weakening ATP binding in the first step of the catalytic cycle.

The analysis of indirect, water-mediated interactions between R324S or R342C and the β- and γ-phosphate groups of ATP, respectively, suggested that the extent by which the R324S mutation weakens ATP binding is smaller than the effect of the R324C mutation. This suggestion was based on three independent but related analyses. First, the visual inspection of computed water density between residue 324 and the β- and γ-phosphate groups of ATP in MD trajectories of the GS_ATP+GLU_ state revealed a much broader region of high water density between R324S and ATP than between R324C and ATP (Fig 2C and 2D). Second, RDFs for oxygen atoms of water molecules around the sidechain oxygen of R324S or sulfur of R324C, respectively, demonstrated a 30% higher water density at the first shell peak for R324S than for R324C, and a considerably more structured second shell in the case of R324S (Fig 2E and 2F). Third, the frequency of occurrence of water-mediated hydrogen bonds between residue 324 and the β- and γ-phosphate groups of ATP is significantly higher for R324S than for R324C for both weak and strong hydrogen bonds (Fig 2G and 2H). Water at the interface of biomolecular complexes can provide increased ligand or substrate affinity [76, 79, 80] due to it mediating the interaction between polar groups *via* hydrogen bonds and/or filling space providing van der Waals interactions [81]. The higher water density, more ordered water structure, and increased number of hydrogen bonds found for R324S compared to R324C is in line with findings that more hydrophilic groups generally lead to a higher number of water molecules at a binding interface [82] and that hydrogen bonds involving sulfur are more elusive and weaker than those involving oxygen [83–86], resulting in a larger hydrogen bonding potential of serine than of cysteine in proteins [83]. Our finding of significantly more frequent water-mediated hydrogen bonds between R324S and the β- and γ-phosphate groups of ATP can thus provide an explanation for the suggestion that GS residual activity is higher in the R324S mutant than in the R324C mutant [20]. This residual activity has been linked with the survival of the now seven-year old boy [20]. As this boy still suffers from hyperammonemia [19], the finding could also stimulate the development of better ATP binding-enhancing molecules by which the R324S mutant can be “repaired” extrinsically [87].

The wild type residue R341 is part of an amino acid triplet located on a loop region including also D339 and R340 [22]. While the sidechain of R341 points away from the catalytic site, R340 points to it (Fig 3A). In the course of our MD simulations, we observed a persistent hydrogen bond of the guanidino function of R340 to GGP in the GS_ADP+GGP_ state (Fig 3B) as also found in the crystal structure [22]. In contrast, for the state GS_ATP+GLU_, we observed a shift of the side chain position of R340 during the MD simulations, leading to a salt-bridge interaction with the γ-phosphate group of ATP (Fig 3B). The loss of salt bridge interactions due to the R341C mutation, observed between R341 and D339 in wild type GS (Fig 3C and 3D), results in a destabilization of R340 in the GS_APO_ state, as manifested by significantly smaller RMSF of that residue in wild type GS than the R341C mutant (Fig 3E). No such changes in R340 mobility were observed for the other three GS states. Hence, this strongly suggests that the R341C mutation indirectly weakens ATP binding in the first step of the catalytic cycle. This suggestion was corroborated by the computed relative effective energy of binding of ATP, which is about 6 kcal·mol^-1^ (equivalent to an approximately 10000-fold lower association constant) less favorable for the R341C mutant than for wild type GS (Fig 6B).

An even more indirect, long-range effect of the R341C mutation on the catalytic efficiency of GS was revealed starting from rigidity theory-based analyses [26, 71] of the structural stability of the GS_ATP_ state. These analyses demonstrated that the loss of side chain interactions in the R341C mutant, particularly to H281, H284, and Y288 on H8, result in a significant destabilization of the C-terminus of GS compared to wild type (Fig 3F). These results were confirmed by the analysis of secondary structure of H8 during MD simulations of wild type GS, the R341C mutant, and the triple alanine mutant of H281, H284, and Y288 (HHY) considered to act as a mimic of the R341C mutant: For the latter two cases, a marked increase in loop probability was found for the central region of H8 (Fig 3G). Together with the above results on R341’s role in the triad, this suggests that R341 can relay stability information between the catalytic site and H8. Remarkably, these results can provide an explanation at the atomistic level as to why addition of ATP to human GS increases the melting temperature of the enzyme by 11.5°C [22].

From comparative crystal structure analysis, a connection between ATP binding in the first step of the catalytic cycle of GS and a shift of H8 that closes GS’ catalytic site was recognized; this shift is considered a prerequisite for glutamate binding and should be hampered in the R341C or HHY mutants [22], which should weaken glutamate binding as suggested by the computed relative effective energy of binding of about 2.2 kcal·mol^-1^ (equivalent to an approximately 40-fold lower association constant) (Fig 6B). We therefore tested the role of amino acids H281, H284, Y288 in human GS for substrate binding to the glutamate binding pocket in HEK293 cells overexpressing HHY-mutated YFP-tagged GS using MSO as a substrate (Fig 4C, 4D and 4E).

MSO binds non-covalently to the glutamate binding pocket of GS and irreversibly inactivates the enzyme [73] thereby masking an epitope which was recently shown to be recognized by a monoclonal antibody raised against GS [62]. In line with this, MSO-treatment strongly reduced anti-GS immunoreactivity in wildtype-YFP-GS transfected HEK293 cells compared to untreated controls (Fig 5A). In contrast, anti-GS immunoreactivity was unchanged in MSO-treated HEK293-cells transfected with HHY-mutated GS, indicating impaired substrate binding to the catalytic center and suggesting loss of enzymatic activity. Mutation of neighboring amino acids S278, K279, and R280, which do not interact with R341 and were suggested to not play a role for maintenance of the tertiary structure of the catalytic center, did not prevent MSO-binding to GS as indicated by reduced anti-GS immunoreactivity (Fig 5A). Reduced anti-GS immunoreactivity in MSO-treated HEK293 cells was not due to MSO-mediated downregulation of YFP-GS in the respective transfected HEK293 cells (Fig 4C).

In order to validate that MSO masks an epitope recognized by the monoclonal anti-GS antibody, protein samples were denaturated, which was shown to release MSO from GS [73] and to recover recognition of GS by the anti-GS antibody [62]. Indeed, this treatment completely restored anti-GS immunoreactivity in MSO-treated HEK293 cells transfected with wild type- and SKR-mutated GS as shown by dot-blot analysis (Fig 5B). In line with this, anti-GS immunoreactivity was unchanged in MSO-treated GS-transfected HEK293 cells compared to untreated controls when analysed by Western-blot using heat- and detergent-treated protein samples (Fig 4D).

Binding of ATP to GS is a prerequisite for accessibility of the active center for glutamate [23, 24]. Thus, impaired glutamate binding as indicated by unchanged anti-GS immunoreactivity in MSO-treated HEK293 cells transfected with HHY-mutated GS (Fig 5A) may be a consequence of impaired binding of ATP. This hypothesis was verified by precipitating ATP-binding proteins from HEK293 cells transfected with wildtype- or HHY-mutated GS by N^6^-(6-aminohexyl)-ATP-agarose and anti-GS Western-blot analysis. Both endogenous and overexpressed wildtype- or HHY-mutated YFP-GS were detected in precipitates of ATP-binding proteins from HEK293 cell lysates (Figure G in S1 file). Almost no anti-GS immunoreactivity was detected when ATP-binding proteins were precipitated in the presence of excess ATP (10 mmol/l; Figure G in S1 file). These data suggest that mutating amino acids H281, H284, and Y288 of GS does not affect the binding of ATP to GS but that of glutamate.

The present findings also confirm earlier results [17] showing that arginine 341 is critical for recognition by a monoclonal antibody (BD, clone6) raised against GS (Fig 4D). In line with this, mutating amino acids H281, H284 and Y288 or S278, K279 and R280 had no impact on GS-recognition by the monoclonal antibody (Fig 4D).

These results suggest that amino acids H281, H284 and Y288 on helix H8 in human GS may stabilize the tertiary structure of the glutamate-binding site through interaction with R341, that way enabling glutamate binding to the catalytic center.

In summary, we identified the molecular mechanisms of the GS mutations R324S, R324C, and R341C that lead to clinically relevant glutamine deficiency. All three mutants are suggested to influence the first step of GS’ catalytic cycle, namely ATP or glutamate binding. Our analyses reveal a complex set of effects including the loss of direct interactions to substrates, the involvement of water-mediated interactions that alleviate part of the mutation effect, and long-range effects between the catalytic site and structural parts distant from it. As to the latter, dot-blot analysis of HEK293 cells overexpressing GS is in line with our prediction of a significant destabilization of helix H8 in the R341C mutant, which should negatively affect glutamate binding.

## Supporting Information

S1 File The file *S1_file*.*pdf* contains additional information to the manuscript: supplemental methods on purification of ATP-binding proteins, distances over the course of the 20–100 ns interval of MD simulations between the side chain of residue 324 and the β-phosphate group of the ATP and/or ADP (Figure A), the mobility of ATP bound to wild type GS and both R324C/S GS mutants (Figure B), time series of distances between the side chain of residue R340 and ATP, glutamate, and ADP (Figure C), results from rigidity analysis of decameric GS expressed as stability map (Figure D), correlation analyses of effective binding energies for ATP (Figure E) and glutamate (Figure F) binding to GS, effect of mutating amino acids H281, H284, and Y288 on binding of ATP to GS (Figure G). (PDF)
