# Supplementary material for: Molecular Mechanisms of Glutamine Synthetase Mutations that Lead to Clinically Relevant Pathologies
Source: PLoS Comput Biol. 2016 Feb 2;12(2):e1004693. doi: 10.1371/journal.pcbi.1004693 (PMC4737493; doi:10.1371/journal.pcbi.1004693)
Supplement: S1 File — (PDF) [file pcbi.1004693.s001.pdf]

Supporting Information

Molecular Mechanisms of Glutamine Synthetase Mutations that Lead to Clinically Relevant Pathologies

Benedikt Frieg[1], Boris Görg[2], Nadine Homeyer[1], Verena Keitel[2], Dieter Häussinger[2$] and Holger Gohlke[1$]

[1]Institute for Pharmaceutical and Medicinal Chemistry, Heinrich-Heine-University, Düsseldorf, Germany

[2]Clinic for Gastroenterology, Hepatology, and Infectious Diseases, Heinrich-Heine-University, Düsseldorf, Germany

[$] Corresponding authors: (H.G.) Universitätsstr. 1, 40225 Düsseldorf, Germany, Phone: (+49) 211 81 13662, Fax: (+49) 211 81 13847, E-mail: gohlke@uni-duesseldorf.de; (D.H.) Moorenstr. 5, 40225 Düsseldorf, Phone : (+49) 211 81 16330, Fax : (+49) 211 81 18752, E-mail : haeussin@uni-duesseldorf.de

Supplemental Methods

Purification of ATP-binding proteins

Prior to purification of ATP-binding proteins, endogenous ATP was removed from HEK293 protein samples using Amicon ® Ultra-4 centrifugal filter devices (Merck/Millipore, Darmstadt, Germany) with a molecular weight cutoff of 10,000 Dalton. ATP-binding proteins were purified from 300 µg of ATP-depleted HEK293 protein samples by precipitation with N^6-(6-Aminohexyl)-ATP-agarose using a commercial kit (ATP AffiPur Kit III, Jena Bioscience, Jena, Germany) according to the instructions of the manufacturer. Precipitates were washed 3 times with washing buffer, and ATP-binding proteins were eluted by incubation of the agarose with elution buffer for 20 min at 4°C. Where indicated, precipitation and washing of ATP-binding proteins with N^6-(6-Aminohexyl)-ATP-agarose was performed in the presence of excess ATP (10 mmol/l).

Supplemental Figures

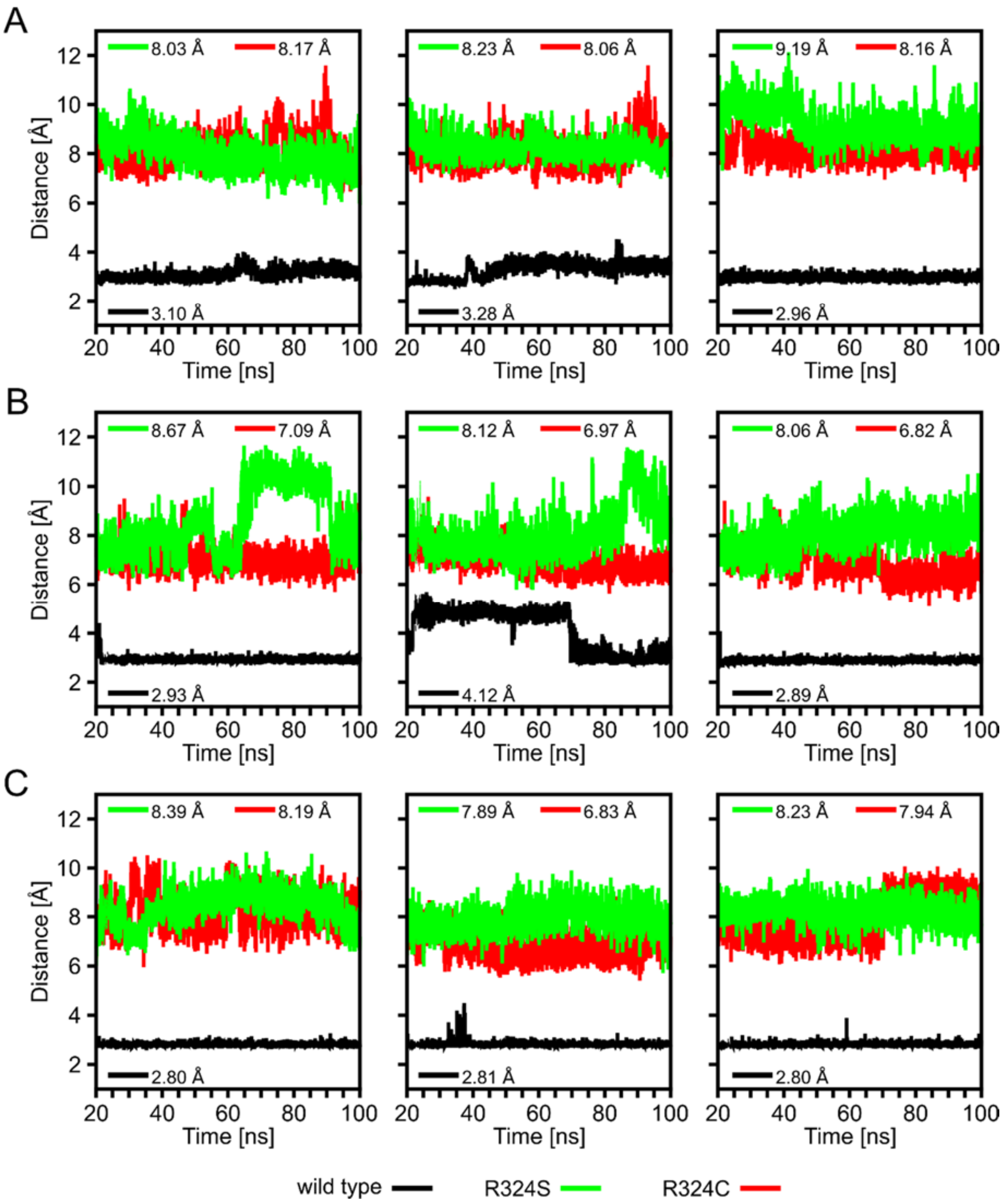

Figure A: Distance between the side chain of residue 324 and the β-phosphate group of the nucleotide.

Distances between R324 (wild type GS; depicted as black lines), or S324 (depicted as green lines) and C324 (depicted as red lines) in GS mutants, and the β-phosphate group of ATP in states GS_{ATP} (**A**) and $GS_{ATP+GLU}$ (**B**) or ADP in state $GS_{ADP+GGP}$ (**C**), respectively, during the 20 – 100 ns interval. The left/middle/right columns show trajectories that were equilibrated at 299.9/300.0/300.1 K, respectively. Mean distances of the respective trajectory are provided in the legend (mean standard error < 0.1 Å).

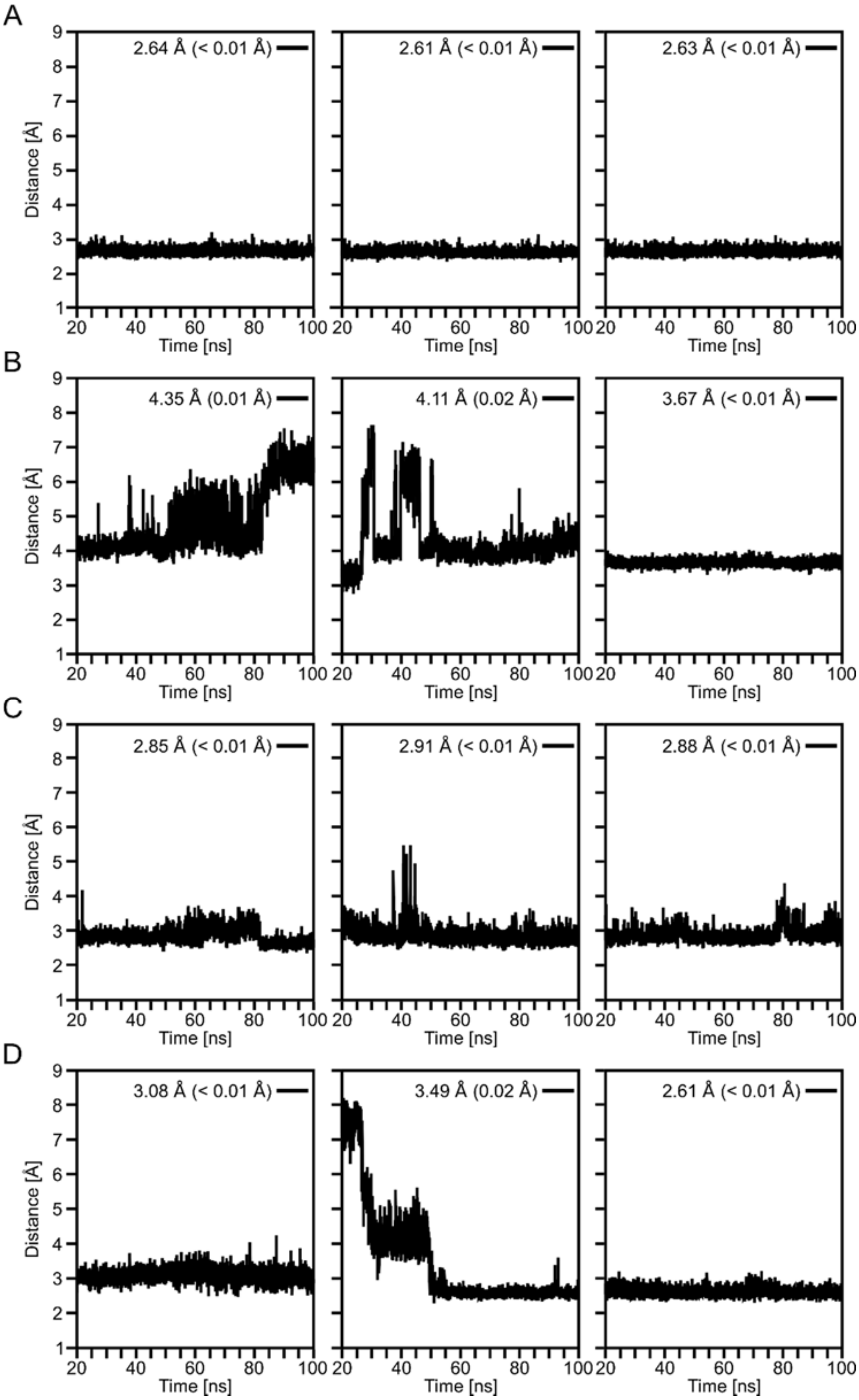

Figure B: Salt bridge interactions between R340 and the substrates.
Distances between R340 and (**A**) the carbonyl oxygen of GGP in state $GS_{ADP+GGP}$, (**B**) the γ-carboxylic function of glutamate in state $GS_{ATP+GLU}$, (**C**) the *γ*-phosphate group of ATP in state GS_{ATP}, and (**D**) the *γ*-phosphate group of ATP in state $GS_{ATP+GLU}$. The left/middle/right columns show trajectories that were equilibrated at 299.9/300.0/300.1 K, respectively. Mean distances of the respective trajectory are provided in the legend with mean standard error in parentheses.

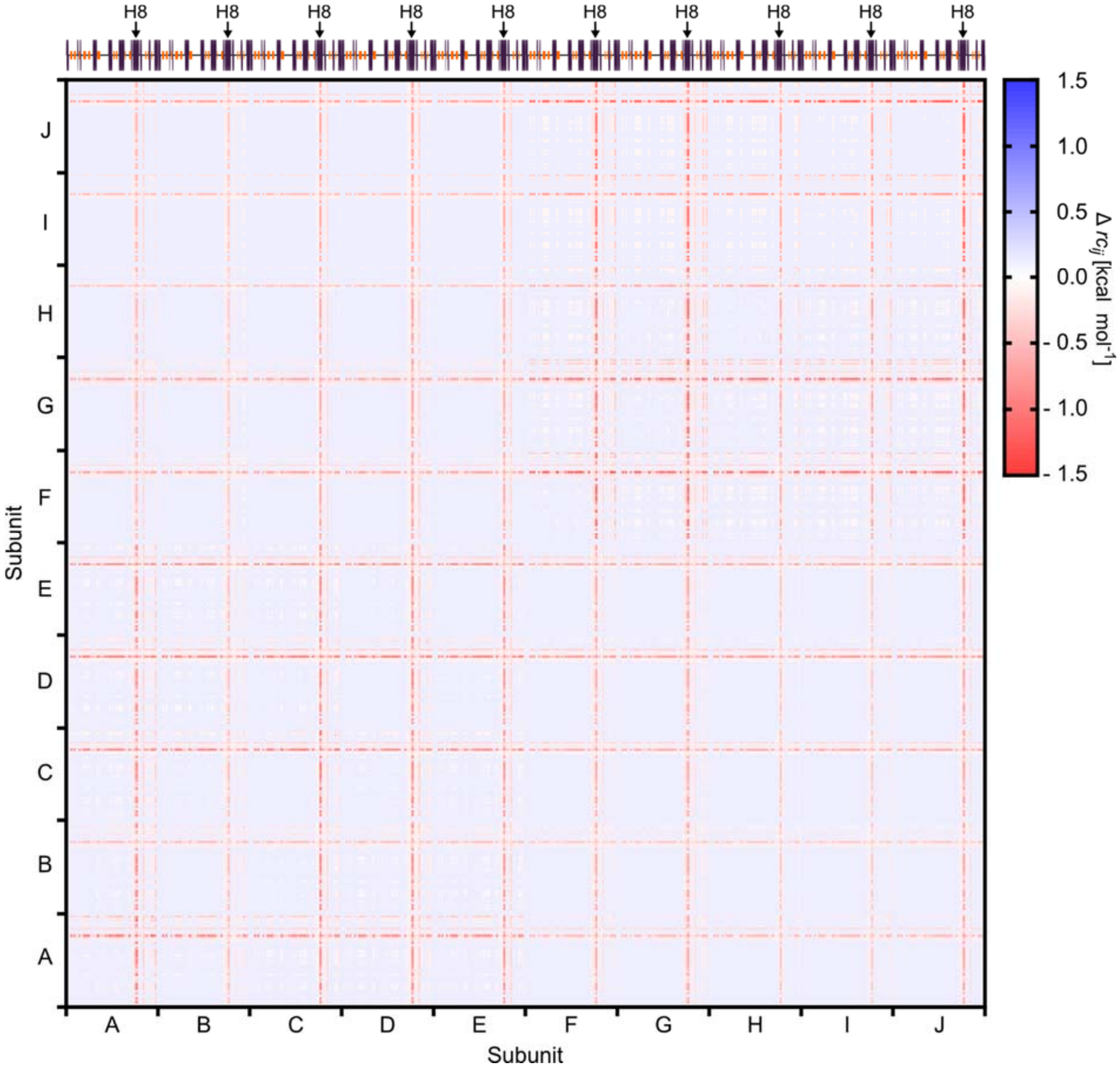

Figure C: Stability changes in decameric GS.
Stability map depicting significant differences ($p < 0.05$) in the structural stability for the GS decamer as computed by CNA between wild type GS and the R341A mutant. Protein structures were extracted from the $GS_{ADP+GGP}$ state. Blue colors indicate that two residues are less stably connected in the wild type, red colors that two residues are less stably connected in the R341A mutant. The secondary structure of GS is depicted on the top, with orange bars representing β-strands and black bars representing α-helices; H8 is labelled.

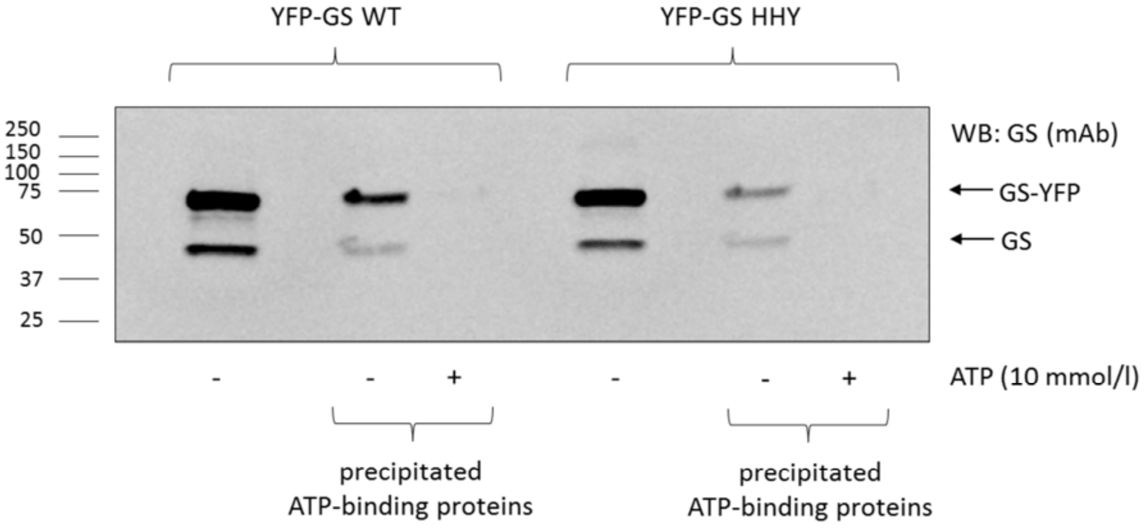

Figure D: Effect of mutating amino acids H281, H284, and Y288 on binding of ATP to glutamine synthetase.
Human embryonic kidney cells (HEK293) were transfected with cDNA constructs coding for YFP-tagged human GS without (WT) or with mutations at positions H281A, H284A, Y288A (HHY). ATP-binding proteins were purified from HEK293 cell protein lysates using N^6AH-ATP-agarose as described in Supplemental Methods, and GS was detected using the monoclonal anti-GS antibody (BD, clone6) by Western-blot analysis. Where indicated, precipitation of ATP-binding proteins was performed in the presence of excess ATP (10 mmol/l). One representative Western-blot out of five is shown.
